# Hsa_Circ_0105596/FTO inhibits progression of Parkinson's disease by sponging miR-187-3p and regulating eEF2

**DOI:** 10.1016/j.heliyon.2024.e39830

**Published:** 2024-11-02

**Authors:** Jiahao Feng, Jin Zhao, Yong Kuang, Yuheng Zhou, Ziheng Ye, Yutong He, Dandan Chen, Li Zhang, Tingying Zhang, Qingqing Zhu, Shumin Cheng, Taoli Liu

**Affiliations:** a Traditional Chinese Medicine Department, The Seventh Affiliated Hospital of Sun Yat-sen University, Shenzhen, 518000, China; b Digestive Disease Center, The Seventh Affiliated Hospital of Sun Yat-sen University, Shenzhen, 518000, China; c Department of Thoracic Oncology, Sun Yat-sen University Cancer Center, Guangzhou, 510632, China; d School of medicine, Sun Yat-Sen University, Shenzhen, 518107, China; e ChangSha Medical University, Changsha, 41000, China; f Scientific Research Center Department, The Seventh Affiliated Hospital of Sun Yat-sen University, Shenzhen, 518000, China; g Shenzhen Key Laboratory of Chinese Medicine Active Substance Screening and Translational Research, Shenzhen, 518000, China

**Keywords:** Parkinson's disease (PD), Hsa_Circ_0105596 (circFTO), miR-187-3p, Transcriptome analysis, Neuronal apoptosis

## Abstract

**Background:**

Parkinson's disease (PD) characterized by inflammation and protein erroneous deposition, whose pathological mechanisms have not been elucidated. NcRNA plays important role in PD, especially when circRNA sponges miRNA, which leads to the breakdown of downstream regulation. The aim of this study is to investigate the dynamic changes between upregulated circRNA and downregulated miRNA during the pathogenesis of PD and their impact on downstream miRNA targets.

**Method:**

We conducted bioinformatics on sequencing data of substantia nigra (SN) and striatum, and intersected differentially expressed genes (Degs) to determine core role of circFTO-miR-187-3p-EEF2 axes in the progression of PD. Firstly, therapeutic effect of knock-down circFTO in PD and its impact on EEF2 were determined in mouse, using immunohistochemistry, HE, Nissl, TUNEL staining and Western blot (WB). Targeted binding relationship between circFTO, miR-187-3p, and EEF2 was determined through RNA binding protein immunoprecipitation assays (RIP) and dual luciferase reporter assay. The significance of gene in apoptosis was confirmed through flow cytometry, lentiviral transduction, quantitative real-time PCR, and WB.

**Result:**

CircFTO is upregulated and miR-187-3p is downregulated in SN of PD. EEF2 was the core of neural repair related modules in both SN and striatum using Weighted Correlation Network Analysis (WGCNA) and protein-protein interaction (PPI). The binding regulation relationship between circFTO-miR-187-3p-EEF2 was determined through structural analysis, RIP, and dual luciferase reporter assay. After knocking-down circFTO, animals showed alleviated symptoms, decreased levels of oxidative stress and EEF2. Upregulation of miR-187-3p or si-circFTO in SH-SY5Y cells can reduce cell apoptosis, EEF2, and oxidative stress. Moreover, individual interference with EEF2 can partially counteract the induction of 6-OHDA on apoptosis.

**Conclusion:**

Excessive circFTO sponging miR-187-3p leads to the inability of miR-187-3p to effectively regulate expression of EEF2, resulting in the progression of PD. Moreover, interference with circFTO can effectively reduce brain inflammation and oxidative stress.

**Table:** Nonstandard Abbreviations and Acronyms

APP	amyloid precursor protein
AS	α-synuclein
circRNA	circular RNA
Cyto c	cytochrome *c*
DAB	diaminobenzidine
Degs	differentially expressed genes
eEF2	Eukaryotic elongation factor 2
eEF2K	Eukaryotic elongation factor 2 kinase
H_2_O_2_	hydrogen peroxide
iPSCs	induced pluripotent stem cells
MDA	Malondialdehyde
ME	Module feature genes
MM	module memory
mRNA	messenger RNA
MUT-	Mutant-
ncRNA	non-coding RNA
NDD	neurodegenerative disease
NF-L	neurofilament light gene
PBS	Phosphate-buffered saline
PD	Parkinson's disease
PPI	protein-protein interaction
qPCR	quantitative real-time PCR
RIP	protein immunoprecipitation assays
rRNA	ribosomal RNA
SN	substantia nigra
SOD	Superoxide dismutase
SS	sterile saline
TH	tyrosine hydroxylase
Thr56	phosphorylates threonine 56
tRNA	transfer RNA
WB	western blot
WGCNA	Weighted Correlation Network Analysis
WT	wild-type
6-OHDA	6-hydroxydopamine

## 1 Introduction

Parkinson's disease (PD) is a neurodegenerative disease (NDD) most common among middle-aged and older people, characterized by clinical symptoms such as tremor, myotonia and bradykinesia [1]. The primary causes of PD include the pathophysiological loss or degeneration of dopaminergic neurons in the substantia nigra (SN), and the formation of neuronal Lewy bodies [2]. Under certain harmful conditions, the cells of substantia nigra degenerate, leading to reduced dopamine synthesis and development of PD [3]. One prior study [3] have shown that eEF2K activity plays a role in α-synuclein (AS) toxicity, and that inhibition of the eEF2K/eEF2 pathway is a potential target for reducing AS-induced oxidative stress in PD. Oxidative stress and inflammation induce neuronal apoptosis, especially those non-coding RNA (ncRNAs), remains limited. Our research aimed to elucidate the regulatory network of ncRNAs in the neural microenvironment.

CircRNAs and microRNAs (miRNAs) are key ncRNAs involved in gene regulation and affect the development of tumors and NDD [4]. Many researchers are committed to identifying circRNAs/miRNAs as biomarkers for disease prediction, diagnosis, and treatment, as they are abundantly expressed in tissues and blood, with stable structures, conserved sequences, and cell- or tissue-specific expression [5]. Our study focused on the relationship between circRNAs and miRNAs, and their role in regulating downstream gene expression. miRNA can regulate gene expression at the post-transcriptional level by inhibiting translation or promoting degradation of messenger RNA (mRNA) [6]. Indeed, our study of the role of miRNAs in PD showed identified significant differential expression of some miRNAs between patients and normal group. These miRNAs were associated with several differentially expressed genes (DEGs), including amyloid precursor protein (APP), AS, Tau protein, and neurofilament light gene (NF-L) [7]. Indeed, the regulation of mRNA expression by miRNA is usually cell/organ-specific and influenced by tissue metabolic state and stress, with miRNA expression levels strongly correlated [8]. As such, miRNA functioning may be hindered by certain substance, while changes in spatial conformation can leads to failure of the regulatory mechanism, rather than inhibiting the production of miRNA. Research has identified a complementary base region between certain circRNAs and miRNAs [9], indicating the possibility of spatial binding between the two. When miRNAs have a circRNA-competitive binding site, the circRNA performs base-complementary pairing with the miRNA, occupying the gene binding site regulated by the miRNA, such that the miRNA cannot bind to th regulatory region elements, resulting in transcriptional regulatory dysfunction. This phenomenon often leads to disease progression.

In our study, we identified cric-FTO (hsa_cir_0105596) and hsa-miR-187-3p, with the target genes EEF2 and EEF2K, respectively, as differentially expressed ncRNAs. In neurons, no factors that are directly antagonistic to the EEF2K/EEF2 pathway have as yet been found to be involved in the regulation of eEF2 phosphorylation, which is why new regulatory factors were searched for in ceRNA networks. Eukaryotic elongation factor 2 (eEF2) and eukaryotic elongation factor 2 kinase (eEF2K) mediate the translocation of peptidyl-tRNA from the ribosomal A site to the P site, thereby facilitating translation elongation and regulating protein synthesis, thus influencing various forms of synaptic plasticity [10,11]. Inhibition of eEF2/eEF2K activity can alleviate amyloid-β (Aβ) oligomer-induced neuronal functional deficits through activation of the NRF2 antioxidant response [12]. Further, inhibition of eEF2/eEF2K activity can reduce the level of reactive oxygen species (ROS) to prevent the associated cellular damage [13]. PD progression has been found to be associated with alterations in the levels of eIF3 and eEF2 in the SN, in a manner associated with SN neuronal loss [14]. This results indicates a key role for eEF2 in the altered machinery of protein synthesis. Another study found that inhibition of eEF2K activity improved mitochondrial function and oxidative damage, while AS accumulation-induced neurotoxicity was alleviated when the EEF2K gene was knocked down [15]. According to the relationship between the substrate and enzyme activity, when the concentration of the substrate eEF2 is low, eEF2K activity is inhibited; therefore, when eEF2 is continuously regulated by miRNA inhibition, the enzyme activity of eEF2K will remain at normal levels in patients with PD. Therefore, based on the interaction between circRNAs and miRNAs, therapeutic tools can be designed to restore the expression or activity of EEF2 and EEF2K to normal levels to treat or cure PD.

## 2 Materials and methods

### 2.1 Raw data

We download mRNA (Striatum [GSE20146, GSE20295, GSE23290, GSE28894 and GSE54282] and SN [GSE20141, GSE20163, GSE20164, GSE24378, GSE26927, GSE42966, GSE49036, and GSE7621]), circRNA (GSE133101) and microRNA (GSE110719) data included in this study were all derived from the GEO database (https://www.ncbi.nlm.nih.gov/geo).

### 2.2 Transcriptome analysis

The original sequencing data (SRA files) of circRNA and mRNA were converted into fastq files using fasterq-dump software, and then cutadapt software was used to remove the adapter sequences from the fastq files, and fastqc software was used to control the quality of the de-linked fastq files. Finally, bwa software was used to align the fastq file to the hg38 reference genome and obtain the sam file. For circRNAs, we used CIRI2 software for circRNA identification and prediction, and DEBKS software for differential expression analysis of circRNAs identified by CIRI2 (|log2FC| > 0.3, FDR <0.05). For mRNAs, we annotated them with featureCounts software and gtf files, and performed differential expression analysis on the expression matrix with the DESeq2 package (|log2FC| >0.3, p.adj <0.05). Based on the R environment, the DESeq2 package was invoked to identify differentially expressed miRNAs (|log2FC| >0.3, p < 0.05). We define these genes as differentially expressed genes (Degs). For multiple datasets, use “sva” and “limma” package to remove batch effects of merging datasets, and present boxplot of the data before and after processing.

### 2.3 GO/KEGG enrichment analysis, GSEA analysis and weighted correlation network analysis (WGCNA)

GO and KEGG enrichment analyses were performed with the aid of R packages “clusterProfiler”, “enrichplot”, and “ggplot2”. Only terms with both p- and q-value of <0.05 were considered significantly enriched. We download the required gmt file from the MSigDB database and perform GSEA analysis on it using the R package “clusterProfiler”.

The “WGCNA” and “flashCluster” packages are used for WGCNA analysis. Firstly, we chose biweight midcorrelation method to construct the adjacency matrix to describe the correlation strength between nodes. Subsequently, select the soft threshold β (striatum 9, SN 6), we transform the adjacency matrix into a topological overlap matrix (TOM). Next, we perform hierarchical clustering to identify modules containing at least 30 genes (minModuleSize = 30), calculate feature genes, perform hierarchical clustering on modules, and merge similar modules. Module feature genes (ME) represent the expression patterns of the module in the sample, while module memory (MM) refers to the correlation coefficient between genes within the module and ME, which is used to determine important clinical phenotype related modules. Finally, we selected modules whose expression changes were consistent with the ND-PD process and extracted core genes for hub gene analysis using node degree and weight analysis via cytoscpe 3.10.0.

### 2.4 Predicting circRNA and miRNA targets

Predict the downstream miRNA targets that hsa_circ_0105596 (circFTO) competitively binds from the circMine bioinformatics website (http://www.biomedical-web.com/circmine/), and evaluate the scores of each downstream target to screen potential miRNAs.

For the potential miRNA predicted by circMine, enter the ENCORI bioinformatics website (https://starbase.sysu.edu.cn/), obtain the potential binding of miRNA to downstream target genes, and sort and determine the potential role of the target in PD.

### 2.5 Establishment of PD model and plasmid injection

All experiments were carried out according to the guidelines of Ethics Committee of the Seventh Affiliated Hospital of Sun Yat sen University (No.202307140). All of the experiments performed in animals were approved by the Francis Crick Institute's Animal Welfare and Ethical Review Body and conformed to UK Home Office regulations under the Animals (Scientific Procedures) Act 1986 including Amendment Regulations 2012. Forty male C57BL/6 mice (10–12 weeks old, weight 23 ± 3 g) were randomly divided into 4 groups (n = 10): Control group, PD group, PD + sh NC group, PD + sh-circFTO group, and were kept at a constant temperature of 24 ± 2 °C and relative humidity of 60 %, with a light dark cycle of 12 h and free access to food and water.

Mice were anesthetized with sodium pentobarbital (40 mg/kg), and 4 μL 6-hydroxydopamine (6-OHDA; H4381, 3 μg/μL, Sigma Aldrich, St. Louis, MO, USA) which was dissolved in sterile saline containing 0.02 % ascorbic acid (SS). PD model was established by stereotaxic injection into the right SN of mice (from bregma: AP, −3.0 mm; ML, −1.2 mm; DV, −4.7 mm), the control group was injected SS [16]. After the mice were resuscitated, penicillin 50,000 U/d was intraperitoneally injected for 7 days to avoid infection. SH-SY5Y cell lines were also modeled by treatment with 6-OHDA.

For knockdown circFTO, we injected targeting shRNA-circFTO lentiviral vector or control vector (pcDNA 3.1, 2 μL, 2.1 × 10^7^ TU/mL) designed and provided by Shanghai Gene Pharma Co., Ltd (Shanghai, China), 0.5 μL/mL min, to the mouse SN region [17], sh-circFTO: 5′- CACCGGCAGAGATCCTGATACTTGGCGAACCAAGTATCAGGATCTCTGCC -3'.

### 2.6 Behavioural test

A week after modeling, the rotation test was induced by intraperitoneal injection of 0.5 mg/kg apomorphine (Sigma-Aldrich, St. Louis, MO, USA). The number of turns of the mouse to the right within 30 min was recorded, and the rotational speed ≥7 r/min was regarded as a successful PD mouse model [18].

### 2.7 Tissue isolation

After the experiment, mice in all groups were euthanized by decapitation before dissection, and then brain tissue was extracted and isolated through craniotomy. Fresh SN tissue was rapidly isolated from the mouse brain with forceps, and the SN tissue was fixed in 4 % paraformaldehyde or stored in liquid nitrogen.

### 2.8 HE staining, Nissl staining and TUNEL staining

SN tissue was embedded in paraffin, cut into 4 μm slices, dewaxed in xylene for 20 min, dehydrated in graded alcohol (100 %, 95 %, 80 %, 75 %) for 1 min, rinsed with distilled water for 2 min, and stained with hematoxylin for 10 min. Sections were treated with hydrochloric acid ethanol for 30 s, soaked in warm water at 50 °C for 5 min, and then stained with eosin for 30 s or toluidine blue dye solution for 5 min. Subsequently, the sections were dehydrated with 70 % and 90 % alcohol for 10 min, permeabilized with xylene, sealed with neutral glue, and the tissue morphology was observed under a microscope. TUNEL detection kit (Roche, USA, 11684795910) was used to process tissue sections and observe stained section under a fluorescence microscope to evaluate the apoptosis of SN regions.

### 2.9 Inflammation index detection and immunohistochemistry

ELISA kits (Multisciences Biotechnology Co., Ltd.) detected SOD, TNF-α, IL-1β content in tissue and cell supernatants, and absorbance was measured at 490 nm using a microplate reader (BioTek, Vermont, USA). Malondialdehyde (MDA) content was detected by MDA content detection kit (Nanjing Jiancheng Bioengineering Institute). Sections were deparaffinized and rehydrated, treated in boiling citrate buffer (pH 6.0) for 15 min, 3 % hydrogen peroxide (H2O2) for 30 min, followed by 0.25 % Triton-X-100 (dissolved in Phosphate-buffered saline (PBS)) permeabilizing for 10 min and incubated with 5 % goat serum (Thermo Fisher, USA) for 1 h at room temperature. Next, anti-tyrosine hydroxylase antibody (AB152, MilliporeSigma) and cleaved caspase-3 (ab2302, Abcam) were incubated with sections at 4 °C overnight, and sections were then incubated with secondary antibody (goat anti-rabbit IgG, #5450-0010, KPL) for 1 h at room temperature. After a final 10 min incubation with 3,3′-diaminobenzidine (DAB) (Thermo Fisher, USA), sections were counterstained with hematoxylin and photographed using a Leica DM1000 microscope (Leica, China).

### 2.10 Cell culture and treatment

SH-SY5Y cells (RRID:CVCL_0019, Cas9X/HyCyte), which was confirmed to be free of contamination using MycoBlue Mycoplasma Detector (vazyme, D101-02), were grown in Dulbecco's Modified Eagle Medium/Nutrient Mixture F-12 (DMEM/F12, C11330500BT, GIBCO) supplemented with 10 % fetal bovine serum (FBS, 10099141C, GIBCO) and 1 % Penicillin-Streptomycin (Pen Strep, 15140-122, GIBCO). Cells were maintained at 37 °C in a saturated humidity atmosphere containing 95 % air and 5 % CO2. Cell dissociation was performed using 0.25 % Trypsin EDTA (25200072, GIBCO), with 1/4 passages per time. Using LipofectamineTM 3000 (Invitrogen, MA, USA), Opti MEM ™ I Reduced Serum Medium (31985070, GIBCO) was transfected with small interfering RNA (siRNA, GenePharma, China) and miR-187-3p inhibitor (GenePharma, China) into SH-sy5y cell line. After transfection, it was replaced with normal medium for 6 h and continued to be cultured for 24 h before being used for subsequent experiments.

### 2.11 Flow cytometry analysis

Inoculate 2.5 × 10^6^ cells per well on 6 well plate, culture for 18 h, then replace the drug containing medium and continue to culture for 24 h. Discard the drug containing culture medium, digest with 0.25 % trypsin without EDTA (15050-065, GIBCO), centrifuge for 1000 rpm, 4 °C, 5 min, wash cells twice with pre-cooled PBS, and resuspend cells with 100 μl Binding Buffer. Add 5 μl Annexin V-FITC and 10 μl PI Staining Solution to tube and gently mix. Avoid light and react at room temperature for 15 min. Add 400 μl of Binding Buffer and mix before placing it on ice. The number of apoptotic cells was assayed by flow cytometry (CogtoFLEX, BECKMAN, USA).

### 2.12 Dual luciferase reporter assay

The wild-type sequence of circFTO or EEF2 and the mutated sequence containing the miR-187-3p binding site were subcloned into the luciferase reporter vector psiCHECK2 (Promega, Madison, WI, USA). They were named WT/MUT-circFTO and WT/MUT-EEF2, respectively. The above luciferase reporter plasmid (50 ng) was co-transfected with miR-187-3p mimic or mimic NC (20 nM) into SH-SY5Y cells using LipofectamineTM 3000. Cells were lysed 48 h after transfection, and the supernatant was collected after centrifugation. Luciferase activity was detected using the Dual-Luciferase Reporter Assay System (E1910, Promega). 100 μL of firefly luciferase working solution was added to each cell sample to detect Firefly luciferase, and 100 μL of Renilla luciferase working solution was added to detect Renilla luciferase. The ratio of renal luciferase activity was used as relative luciferase activity. When miR-187-3p is able to bind to the inserted sequence (circ-FTO or EEF2) and inhibit the translation of firefly fluorescent protein, resulting in a decrease in fluorescence value, we use corrected fluorescence intensity to determine the binding efficiency of miR-187-3p with circ-FTO/EEF2.

### 2.13 RNA immunoprecipitation experiment

The AGO1 and AGO2 binding sites were acquired from published photoactivatable cross-linking immunoprecipitation (PAR-CLIP) data in GEO database (https://www.ncbi.nlm.nih.gov/geo/). We analyzed the AGO1 as well as AGO2 binding sites of hsa_circ_FTO genomic region, and select anti Ago2 antibody for subsequent experiments.

RNA Binding Protein Immunoprecipitation Assays (RIP) were performed using the EZ-Magna RIP kit (Millipore, Billerica, MA, USA). SH-sy5y cells were lysed using RIP lysis buffer (Millipore, Billerica, MA, USA) containing protease and RNase inhibitors, and the RIP lysate was mixed with RIP containing magnetic beads conjugated to human anti-Ago2 antibody or nonspecific mouse IgG antibody (Millipore) buffers were incubated together. Digestive precipitation with protease K and quantitative real-time PCR (qPCR) analysis of immunoprecipitated RNA to detect RNA enrichment. We used the Percentage Input (% Input) method for RIP enrichment level analysis. Calculation method: ΔCt [normalized RIP] = (Ct [RIP] - (Ct [Input] -Log2(Input Dilution Factor)), Input Dilution Factor (IDF)= (fraction of the Input saved) −1, ΔΔCt [RIP/IGg] = ΔCt [normalized RIP] - ΔCt [IgG], %Input = 2 ˆ (CtInput-CtRIP) × IDF × 100 %, Fold Enrichment = 2ˆ (-ΔΔCt [RIP/IgG]).

Primers used in this study were listed as follows: hsa_circ_0039397 (circFTO, human), forward: 5′- TACAACGCTGTCAGTTGGCT-3′, reverse: 5′- TCCCTGCCTTCGAGATGAGA-3′, mmu_circ_0001715 (circFTO, mouse), forward: 5′- CCATAATGAGGCTCTGAGGATGA -3′, reverse: 5′- CAGCCAAAACACAGTGCTGG-3′, EEF2 (human), forward: 5′- GCCTCATGGAGCCCATCTAC-3′, reverse: 5′- AGGGATGCCTTCTTTCAGGC-3′, miR-187-3p, forward: 5′-CACAGGACCCGGGCG-3′, reverse: 5′-CCGGCTGCAACACAAGAC-3′, U6, forward: 5′-CTCGCTTCGGCAGCACA-3′, reverse: 5′-AACGCTTCACGAATTTGCGT-3′, GAPDH (human), forward: 5′- CACCCACTCCTCCACCTTTG-3′, reverse: 5′- CCACCACCCTGTTGCTGTAG -3′, GAPDH (mouse), forward: 5′-CATCAACGGGAAGCCCATC-3′, reverse: 5′- CTCGTGGTTCACACCCATC -3'.

### 2.14 Western blot

Briefly, brain or SH-SY5Y cells were lysed using RIPA buffer containing a complete protease inhibitor cocktail (Roche). Protein concentrations were determined using a BCA protein assay kit (Vazyme). Proteins were then electrophoresed in SDS–PAGE and transferred on a PVDF membrane (Merck millipore). After incubation in 5 % non-fat milk, membranes were immunoblotted with the following antibodies: Elongation factor 2 (EEF2, Abcam, human/mouse), Cytochrome *c* (Cyto C, Cell Signaling Technology, human/mouse), Bax (Cell Signaling Technology, human/mouse), Bcl-2 (Abcam, human/mouse), cleaved-caspase-3 (Abclonal, human/mouse), β-actin (Abcam, human/mouse).

### 2.15 Single-cell RNA-seq analysis

Single-cell RNA sequencing data from midbrain samples of 5 PD patients and 6 controls were downloaded and reanalyzed from the original study (GSE157783). The expression matrix was converted into a Seurat object following the protocol outlined in “Seurat” package. Subsequently, normalization using the NormalizeData function and centered using ScaleData function. Principal component analysis (PCA) was performed with the RunPCA function, selecting the first 20 principal components for UMAP and t-SNE to facilitate dimensionality reduction and clustering analysis. Clusters were meticulously annotated based on gene expression profiles and visualized using integrated tools within “Seurat” package. Statistical analysis was mainly carried out by R (4.1.3), and the two-tailed Student's *t*-test was used to compare groups. A p-value less than 0.05 was considered statistically significant.

## 3 Results

### 3.1 Identification and characterization of key modules in the substantia nigra and striatum in patients with PD

**Fig. 1:**
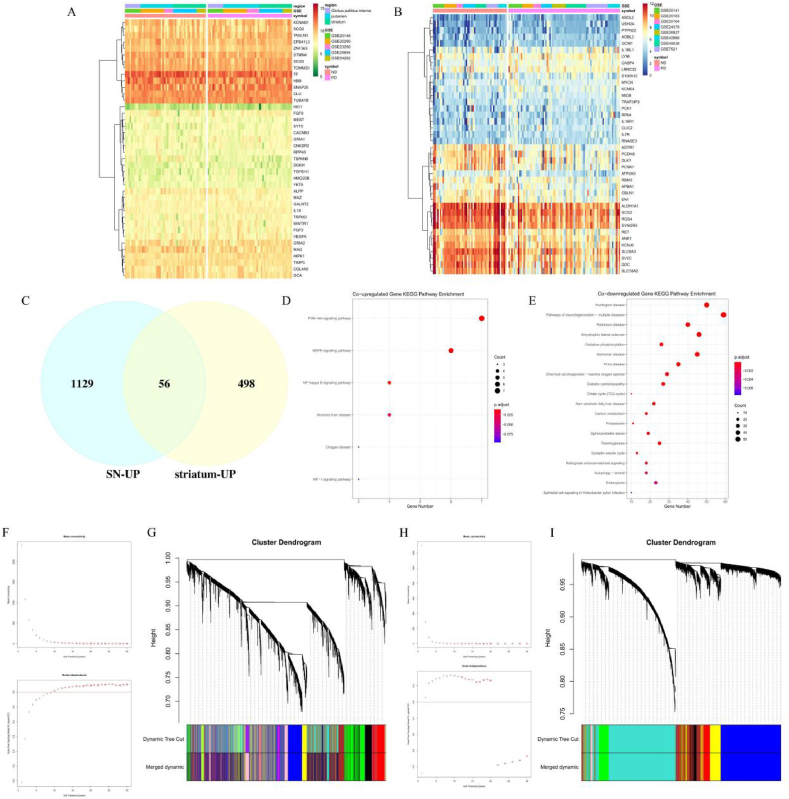
| Identification of expression levels of phenotype related modules and hub genes in Parkinson's disease. A. Heatmap of the top Degs in the normal and PD's striatum; B. Heatmap of the top Degs in the normal and PD's SN, the row represents the gene name, the column represents a sample, the vertical color band represents the expression intensity, and the horizontal color band represents the tissue source, gse number, and symbol, respectively; C. The up-regulated Degs intersect in the SN and striatum, blue represents up-regulated Degs in the SN, while yellow represents up-regulated Degs in the striatum; D. Visualization of KEGG enrichment analysis of co-upregulated Degs; E. Visualization of KEGG enrichment analysis of co-downregulated Degs, size of the dot represents the number of enriched Degs and the color band represents the p.adjust value; F. Analysis of the scale-free index for various soft-threshold powers (β) and Analysis of the mean connectivity for various soft-threshold powers in striatum; G. Dendrogram of all striatum Degs clustered based on the measurement of dissimilarity (1-TOM); H. Analysis of the scale-free index for βand soft-threshold powers in SN; I. Dendrogram of all SN Degs clustered, different colors represent different functional modules.

**Fig. 2:**
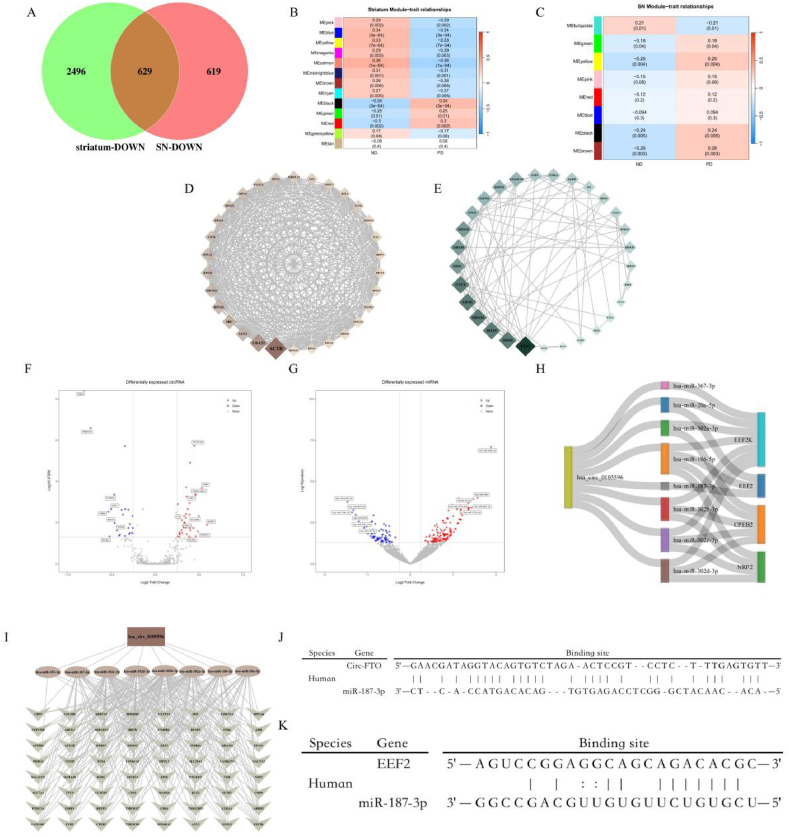
| Identify the relationship ce-network of the core pathogenic factors of Parkinson's disease. A. The down-regulated Degs intersect in the SN and striatum, red represents down-regulated Degs in the SN, while green represents down-regulated Degs in the striatum; B. Heatmap of the correlation between the striatum module eigengenes and clinical traits of PD; C. Heatmap of the correlation between the SN module eigengenes and clinical traits of PD; D. Hub gene network analysis of the striatum phenotype related module MEbrown; E. Hub gene network analysis of the SN phenotype related module MEturquoise, the size of the gene block represents the number of nodes, and the color represents the source module; F. Volcano plots of circRNA in the SN of PD; G. Volcano plots of miRNA in the SN of PD, the horizontal axis represents logFC, the vertical axis represents FDR, the blue dot represents down-regulated Degs, the red dot represents up-regulated Degs, and the gray represents non-Degs; H. Sankey plot of circFTO, miRNA, and their core downstream targets; I. Network topology analysis of downstream targets of ceRNA in circFTO; J. Base complementary pairing diagram of circFTO and miR-187-3p; K. Base complementary pairing diagram of EEF2 and miR-187-3p, vertical lines represent hydrogen bonds of base pairs.

Since changes in the SN and striatum are at the core of PD, we analyzed transcriptome sequencing data from both the SN and striatum. Using the normal striatum as a control, 554 and 3125 up and downregulated genes in the PD group were identified (Supplementary Table 1). In the SN, with ND as the control, there were 1185 and 1248 up and downregulated genes were similarly identified in the PD group (Supplementary Table 2). In ND-PD of the striatum (Fig. 1A), the most significant downregulated DEG was ALPP (logFC = −1.48, p.adj<0.05), and the most upregulated DEG was TIMP3 (logFC = 0.90, p.adj<0.05). In ND-PD of the SN (Fig. 1B), the most significant DEGs were SLC18A2 (logFC = −1.85, p.adj<0.05) and S100A12 (logFC = 1.23, p.adj<0.05). In the SN and striatum, 56 genes were co-upregulated (Figs. 1C) and 629 genes were co-downregulated (Fig. 2A). The results of KEGG enrichment analysis on co-DEGs revealed that the co-upregulated genes were primarily enriched in the PI3K-Akt, MAPK, NF kappa B, and HIF-1 signaling pathway (Fig. 1D), while the co-downregulated genes were enriched in Huntington's disease, neurogenesis pathways, PD, Alzheimer's disease, citate cycle, and thermogenesis (Fig. 1E). We subsequently constructed a gene module network using WGCNA, identifying PD-related modules with a soft threshold power set to 9 and 6 and a height cutoff set to 140, which excluded outlier samples. Due to the scale independence reaching 0.9 and high average connectivity (Fig. 1F and H), in subsequent analysis, the soft threshold powers of the striatum and SN were set to 9 and 6 respectively. Finally, 13 striatal and 8 SN gene co-expression modules were constructed for subsequent analysis (Fig. 1G and I).

### 3.2 Construction of phenotype-ceRNA networks in PD

We subsequently conducted a correlation analysis between the corresponding modules in the striatum and SN, with phenotype. Fig. 2B and C shows the correlation and significance of the modules and phenotypes, respectively. Supplementary Table 3 displays the genes and functions of the main modules, among which the MEbrown module was most strongly related to PD in the striatum, and was enriched in nerve growth and repair. The menturquoise module was predominantly enriched in pathways related to nerve regeneration in the SN. As shown in Fig. 2D, network analysis was performed on the hub genes of the MEbrown module, with the top 3 genes being ACTB, UBA52, and EEF2. As shown in Fig. 2E, the most important gene in the turquoise module was EEF2, indicating that EEF2 may be a key factor in PD. To investigate the regulatory mechanism of EEF2, we conducted a differential expression analysis of circRNAs and miRNAs in the SN. Using ND as control (Figs. 2F), 53 different circRNAs were identified in the ND-PD group, of which 31 were upregulated and 22 were downregulated. In the ND-PD group, the most significantly downregulated circRNA was FMN2 (logFC = −0.81, FDR<0.05), while the most upregulated circRNA was SHPRH (logFC = 0.59, FDR<0.05). Using ND as a control (Figs. 2G), 132 and 78 up- and downregulated miRNAs were identified in induced pluripotent stem cells (iPSCs) of the PD group. In neurons, 104 and 168 up- and downregulated miRNAs were identified.

circRNAs sponge miRNAs in a process which abrogates the regulatory mechanism of miRNAs on mRNA. Therefore, we investigated up/downregulated circRNAs in the PD group, with corresponding down/upregulated miRNAs in the PD group, to investigate possible interactions between them. Fig. 2H and I shows reasonable targeting relationships and target networks for each group. We selected miR-187-3p related to circFTO and EEF2 for further analysis, as this miR-187-3p showed consistent trends compared to other miRNAs in iPSCs (logFC = −0.346) and neurons (logFC = −0.346), which were all derived from the healthy group and PD patient biopsies. Supplementary Tables 4 and 5 show the predicted binding related parameters. Based on the predictive tools, we performed spatial structure fitting of circFTO, miR-187-3p (Fig. 2J), miR-187-3p, and EEF2 (Fig. 2K). The complementarity of the bases indicates possible spatially targeted binding relationships.

### 3.3 Identification of the potential relationship between EEF2, circFTO, and cell apoptosis in animal models

**Fig. 3:**
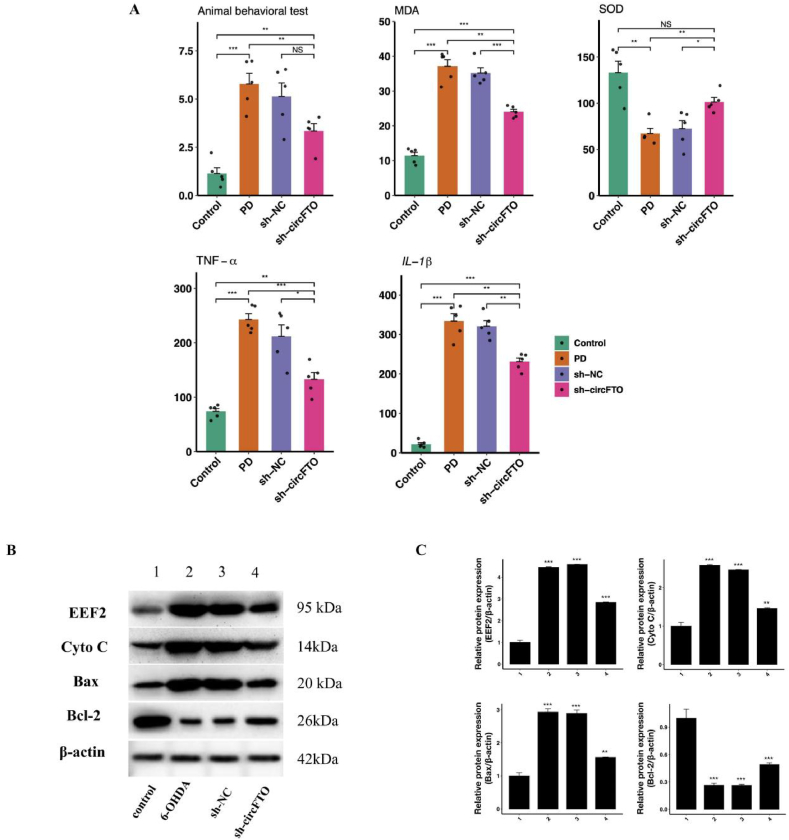
| Exploring the relationship between animal models of Parkinson's disease and protein level of EEF2. A. The box plot shows animal behavior experiment and the levels of SOD, MDA, TNF-a, and IL-b in brain tissue at different treatment groups, different colors represent different processing groups; B. Western blot showed the protein levels of EEF2, Cyto c, Bax, Bcl-2, and β-actin, the left row represents the protein name, the right represents the protein kDa, and the column represents the treatment group; C. The bar chart shows ratio differences in the protein level of EEF2/β-actin, Cyto c/β-actin, Bax/β-actin and Bcl-2/β-actin.

In the animal rotation experiment, compared to the 6-OHDA group, the symptoms of the sh-circFTO group were significantly improved (p < 0.05). Following sh-circFTO intervention in the brain, the levels of oxidative stress indicators SOD and MDA both decreased (p < 0.05) compared with 6-OHDA group, as did the inflammatory factors TNF-α and IL-1β, indicating an improvement in the neural microenvironment. As shown in Fig. 3A*,* after injection of sh-circFTO, the levels of oxidative stress and inflammatory factors decreased, indicating that sh-circFTO can hinder neuronal apoptosis by inhibiting these indicators. The WB results (Fig. 3B) and quantitative analysis (Fig. 3C) further showed that the expression of EEF2 was the lowest in the control group, while expression in the 6-OHDA and sh-NC groups was higher than that in the other groups, and decreased in sh-circFTO group. There was no significant difference in cytochrome *c* (Cyto c) levels between the 6-OHDA and the sh-NC groups, indicating a higher level of apoptosis. However, the level of Cyto c in the sh-circFTO group decreased, indicating a decrease in apoptosis. Following sh-circFTO treatment, the level of Bax was found to be between that in the control and 6-OHDA groups, indicating that sh-circFTO can inhibit apoptosis. Bcl-2 inhibited cell death, while WB results showed that the Bcl-2 levels increased after treatment with sh-circFTO.

**Fig. 4:**
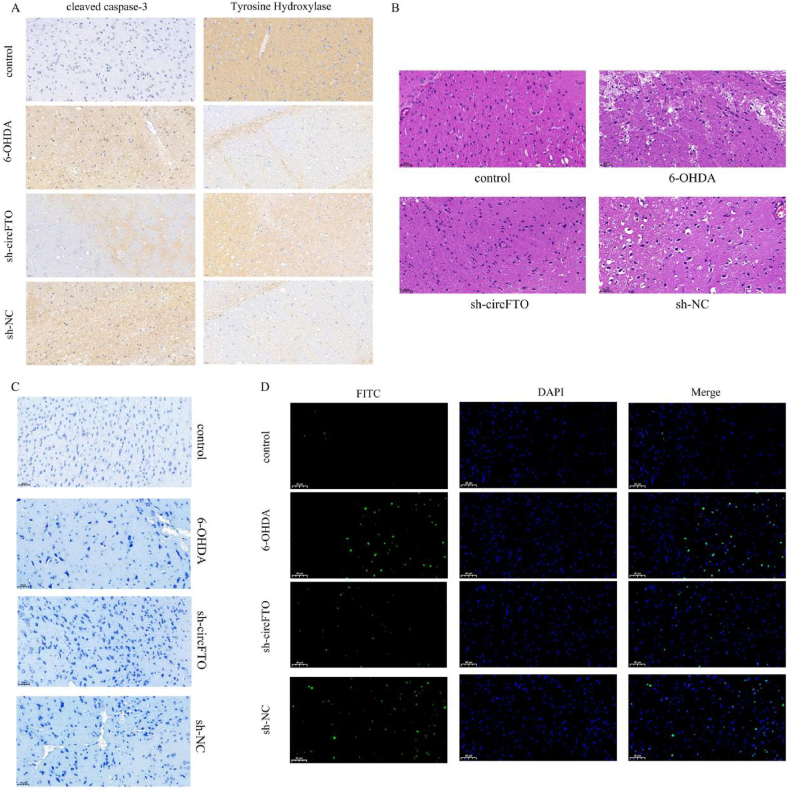
| The substantia nigra section of the Parkinson's disease model reveals the role of circFTO. A. Immunohistochemical assessment of Tyrosine Hydroxylase and cleaved caspase-3 positive cells in the SN of mouse brain; B. HE staining to assess neuronal damage in the SN of mouse brain; C. Nissl staining to assess neuronal damage in the SN of the mouse brain; D. TUNEL staining to assess the rate of neuronal apoptosis in the SN of mouse brain.

Insufficient tyrosine hydroxylase (TH) expression directly affects dopamine synthesis and secretion, while cleaved caspase-3 is a predictor of neuronal apoptosis. Disorders of dopamine synthesis and secretion in dopaminergic neurons act as direct pathogenic factor in PD progression, while TH and cleaved caspase-3 can indicate PD recovery. Compared to the control group, the expression of cleaved caspase-3 was increased and the expression of tyrosine hydroxylase was decreased in the model group, indicating that neuronal apoptosis was increased and dopamine synthesis was deregulated in the 6-OHDA model. However, these changes in the levels of cleaved caspase-3 and TH in 6-OHDA mice were reversed following administration of the sh-circFTO plasmid (Fig. 4A). Nissl staining results (Fig. 4C) showed that the number of cells in the control group was normal, while the number of Nissl bodies was high, indicating that neuronal protein synthesis was active, whereas the 6-OHDA and the sh-NC groups showed a decrease in the number of cells, with a more sparse arrangement, and a small number of Nissl bodies, indicating nerve cells damage, resulting in dysfunction of protein synthesis. After circ-FTO expression was intervened in the sh-circFTO group, the cells tended to be neatly arranged, while the number of Nissl bodies increased, indicating that the damaged cells regained their vitality. The results of HE staining (Fig. 4B) also showed that sh-circFTO had a positive effect. TUNEL staining (Fig. 4D) further showed that the proportion of TUNEL-positive cells in the normal group was very low, while the proportion of TUNEL-positive cells in the model group was the highest; following lentivirus transfection, the proportion of TUNEL-positive cells in the sh-circFTO group began to decrease, while the proportion of TUNEL-positive cells in the model group was the highest.

### 3.4 Identifying the regulatory network of ceRNA

**Fig. 5:**
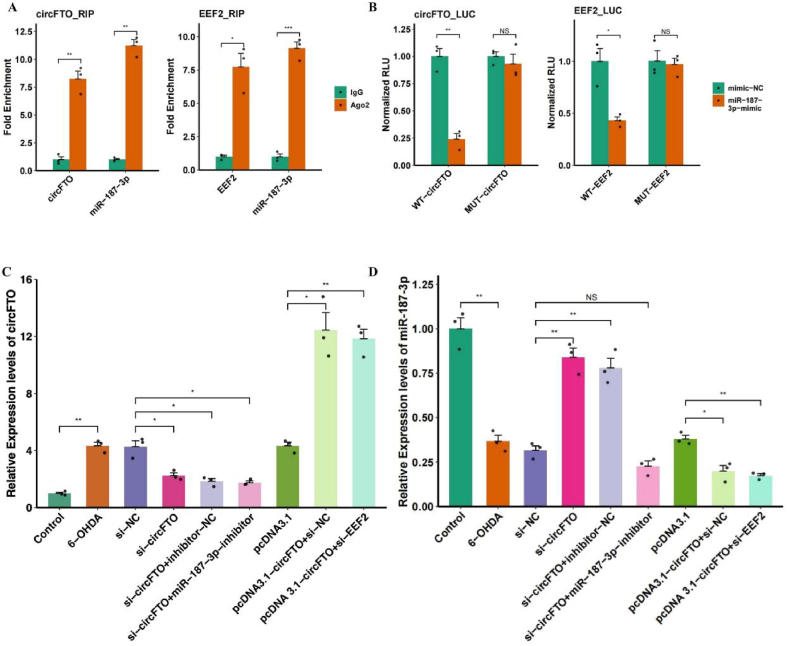
| Identify the interaction between circFTO, miR-187-3p, and EEF2. A. Validation of circFTO-to-miR-187-3p and EEF2-to-miR-187-3p interactions by RNA immunoprecipitation, Ago2 represents the ability of miRNA binding proteins to precipitate lncRNA; B. Validation of circFTO (WT/MUT)-to-miR-187-3p and EEF2 (WT/MUT)-to-miR-187-3p interactions by dual-luciferase reporter assay, MUT represents mutated gene, while WT represents a wild-type gene, used to demonstrate the possibility of binding; C. Real-Time PCR (qPCR) to assess the expression levels of circFTO in SH-SY5Ycells; D. Real-Time PCR (qPCR) to assess the expression levels of miR-187-3p in SH-SY5Y cells, the horizontal axis represents different treatment groups, and the vertical axis represents gene expression levels.

To determine the exact relationship between circFTO, miR-187-3p, and EEF2, we validated their binding using a dual luciferase assay and RIP. Fig. 5A shows the binding relationship between miR-187-3p and circFTO, and between miR-187-3p and EEF2. Ago2 antibody was capable of pulling down miRNA, while we observed a difference in the expression level of miR-187-3p between the IgG-negative group and the ago2 pull down group (p < 0.05), indicating a reasonable efficiency of Ago2 in pulling down miR-187-3p. In quantitative analysis of miR-187-3p pulling down RNA, the relative expression levels of circFTO and EEF2 in the Ago2 group were significantly higher than in the IgG group (p < 0.05), indicating that miR-187-3p could bind to circFTO and EEF2. A dual luciferase assay was applied to compare the fluorescence intensity of mutant EEF2 (MUT-EEF2) and mutant circFTO (MUT-circFTO), with that of the wild-type (WT). The results showed that the fluorescence intensity of miR-187-3p was significantly lower in WT-EEF2 compared to the NC (p < 0.05), while there was no difference between the two groups in the MUT-EEF2 group, indicating that miR-187-3p binds to the 3′UTR of EEF2 to inhibit luciferase transcription. Similarly, miR-187-3p also bound to circFTO to inhibit luciferase expression (Fig. 5B, p < 0.05).

RT-qPCR (Fig. 5C and D) in different in vitro treatment groups (6-OHDA, control, pcDNA 3.1, pcDNA 3.1-circFTO + si-EEF2, pcDNA 3.1-circFTO + si-NC, si-circFTO, si-circFTO + inhibitor-NC, si-circFTO + miR-187-3p-inhibitor, si-NC) showed that the relative expression level of circFTO was higher in the 6-OHDA group than in the ND group, while the relative expression level of miR-187-3p was lower in the 6-OHDA group than in the ND group, indicating that high expression of circFTO in the PD in vitro model may lead to a decrease in the surface expression of miR-187-3p due to the circFTO sponging action on miR-187-3p. Similarly, circFTO overexpression in the pcDNA 3.1-circFTO + si NC group resulted in a significant decrease in miR-187-3p expression compared to that in the 6-OHDA group, indicating that miR-187-3p was sponged by circFTO. However, in the si-circFTO group, the expression of miR-187-3p gradually recovered, which may be related to the improvements in the phenotype of PD observed in the in vitro models following injection with circFTO.

### 3.5 CeRNA network and inflammatory and oxidative stress processes

**Fig. 6:**
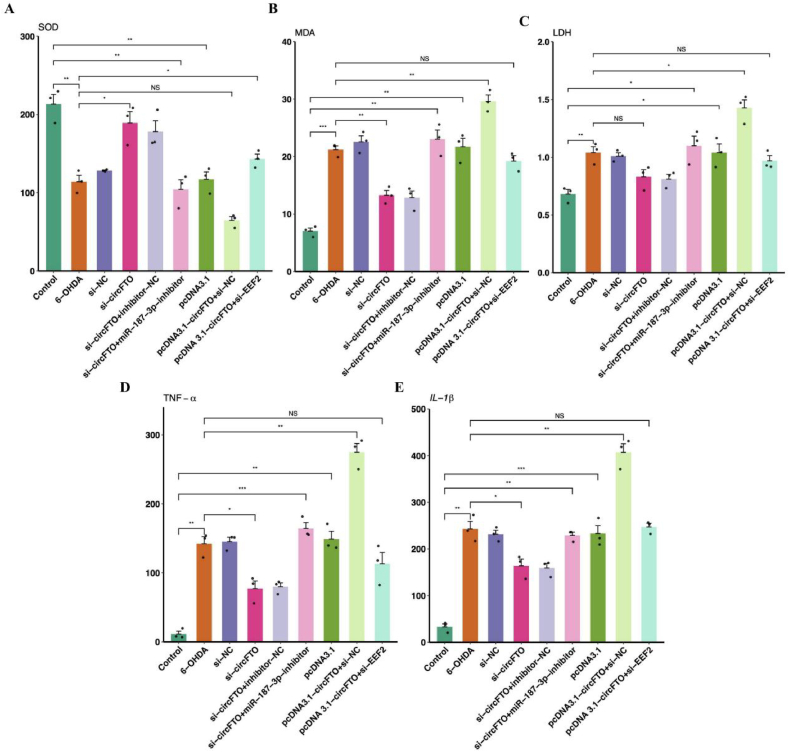
| Regulatory network in circFTO, miR-187-3p, and EEF2. A. SOD levels in SH-SY5Y cells of different treatment groups; B. MDA levels in SH-SY5Y cells of different treatment groups; C. LDH levels in SH-SY5Y cells of different treatment groups; D. TNF-α levels in SH-SY5Y cells of different treatment groups; E. IL-1β levels in SH-SY5Y cells of different treatment groups; different colors represent different processing groups, and the vertical axis represents measured values.

We measured the levels of the oxidative stress indicators SOD, MDA, LDH, and the inflammatory indicators TNF, IL-1β in different treatment groups, as 6-OHDA caused oxidative stress and mitochondrial dysfunction. Comparison of SDO levels (Fig. 6A) showed a significantly lower level in the 6-OHDA group compared with in the ND group (p < 0.05). However, the value of SOD increased compared to that in the 6-OHDA group after interfering with circFTO (p < 0.05). However, in the si-circFTO + miR-187-3p inhibitor group, the SOD level was lower than that in the ND group (p < 0.05), indicating that the regulation of downstream genes by miR-187-3p is a core function of the ceRNA regulatory network. Similarly, the MDA levels showed the same trend (Fig. 6B). As showed in Fig. 6C, the pcDNA 3.1 circFTO + si-NC group showed a significant increase in LDH levels compared to the 6-OHDA group after circFTO overexpression (p < 0.05). Regarding the inflammatory factor (Fig. 6D), the si-circFTO group showed a significant decrease in TNF-α levels compared to 6-OHDA group (p < 0.05). Between the si-circFTO + inhibitor NC and si-circFTO + miR-187-3p inhibitor groups, it was demonstrated that miR-187-3p affects TNF-α levels, but it is significantly lower in the si-circFTO group than in the 6-OHDA group (p < 0.05). After overexpression of circFTO, the TNF-α level was significantly higher than that of the 6-OHDA group (p < 0.05). The trends in IL-1β levels in each group were also similar to TNF-α (Fig. 6E). However, it is worth noting that neither the upregulation of circFTO, nor the interference with EEF2 showed any significant improvement in the inflammatory indicators.

### 3.6 CricFTO regulates apoptosis via EEF2

**Fig. 7:**
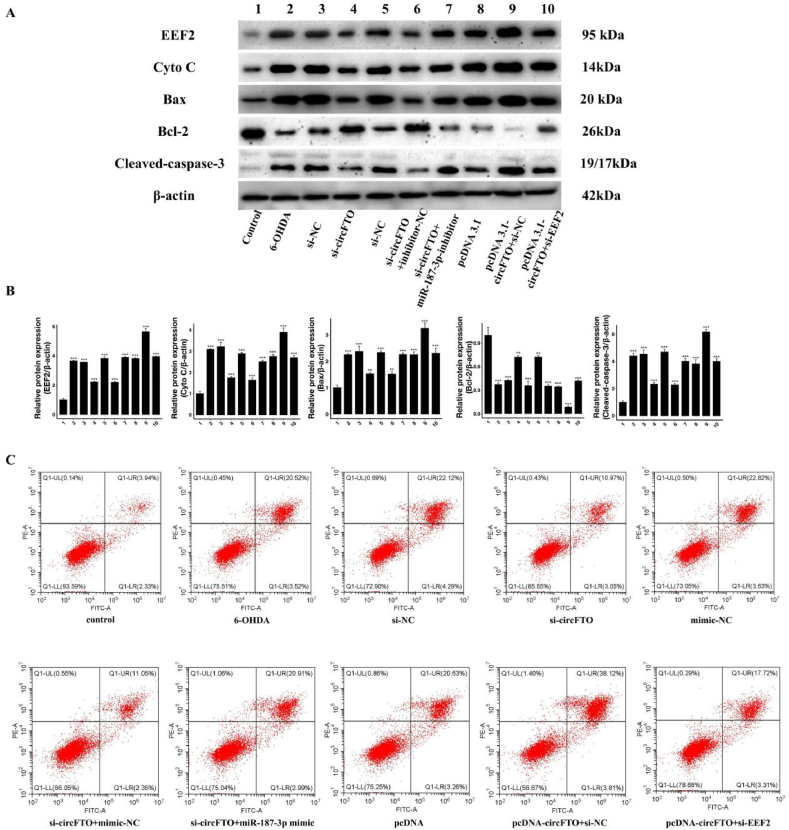
| EEF2 is a downstream target of the interaction between circFTO and miR-187-3p. A. Western blot showed the protein levels of EEF2, Cyto c, Bax, Bcl-2, and β-actin in different treatment SH-SY5Y cells; B. The bar chart shows ratio differences in the protein level of EEF2/β-actin, Cyto c/β-actin, Bax/β-actin and Bcl-2/β-actin; C. Evaluation of SH-SY5Y cells apoptosis levels by flow cytometry with Annexin V and PI.

The results (Fig. 7A and B) showed that the protein levels of EEF2, Cyto c, Bax2, and cleaved caspase-3 in the si-circFTO group (Group 4) and si-circFTO + inhibitor NC group (Group 6) were lower than those in the model group, indicating that interference with circ-FTO can inhibit the expression of EEF2 and cell apoptosis. The proteins of EEF2, cytoc, bax2, and cleaved caspase-3 were all highest in the pcDNA 3.1 circFTO + si-NC group (Group 9), while the expression level of bcl-2 was the lowest, indicating that high expression of circFTO induced cell apoptosis, even more strongly than in the 6-OHDA group. The pcDNA 3.1 circFTO + si-EEF2 group (Group 10) showed that interference with EEF2 could achieve partial functional recovery, which could explain why the improvement in inflammatory and oxidative stress indicators was not significant in Group 10, as si-EEF2 may only partially offset the functional loss of miR-187-3p. Flow cytometry (Fig. 7C) further revealed decreased apoptosis following si-circFTO treatment, with no difference in the apoptosis rate between the si-circFTO + miR-187-3p mimic group (20.91 % + 2.99 %) and the 6-OHDA group (20.52 % + 3.52 %), indicating that miR-187-3p functions as a regulatory mechanism hub. Overexpression of circFTO, increased the apoptosis rate (38.12 % + 3.81 %); however, the application of si-EEF2 decreased the apoptosis rate of SH-SY5Y cells (17.72 % + 3.31 %), indicating that reducing the expression of EEF2 could inhibit neuronal apoptosis.

### 3.7 Single cell analysis validates expression of EEF2/EEF2K in PD neurons

**Fig. 8:**
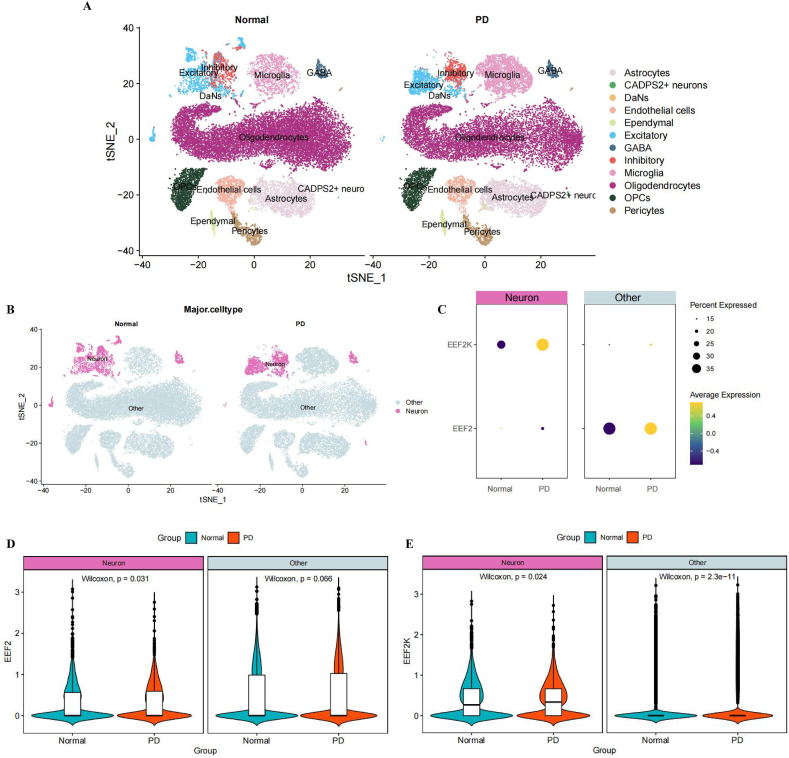
Single cell landscape of EEF2/EEF2K in brain. A. T-distributed stochastic neighbour embedding (tSNE) plots of PD and normal donors, different colors represent different clusters; B. Distribution of neuron and other types in PD and normal; C. Dotplot of neuron and other types in PD and normal, boxes represent cell types, colors represent expression levels, and dot sizes represent percentages; D. Violin plot of EEF2 expression levels in neuron and other types; E. Violin plot of EEF2K expression levels in neuron and other types, different colors represent different groups.

Fig. 8A shows the main landscape of the neural microenvironment in patients with PD and the normal population, showing that neurons are predominantly composed of GABA and dopaminergic neurons. Analysis of neurons in the PD and ND groups revealed that the PD group was able to capture more CADPS2+neurons. Another study found that high CADPS2 expression is a neuronal marker of idiopathic PD [19]. We divided the cells in the cellular landscape into neuronal and other types, and compared their expression differences between different cell types on EEF2 and EEF2K (Fig. 8B). Fig. 8C further shows the expression intensity of EEF2/EEF2K in neurons and other tissues. The expression intensity of EEF2/EEF2K was higher in the PD group than in the normal group. The figure shows that the expression level of EEF2 in the PD group was significantly higher than that in the ND group in neurons (p = 0.031 < 0.05), while there was no difference between the other groups (p = 0.066 > 0.05). EEF2K expression was also significantly higher in other neurons in the PD group compared with those in the normal group (p < 0.05).

## 4 Discussion

The pathogenic mechanisms of PD, a common NDD, involves pathways involving the genome, transcriptome, and proteome, making the development of therapeutic drugs difficult. circRNAs and miRNAs have both been shown to impact cellular function, acting as important components of the epigenetic regulatory mechanisms. Specifically, miRNAs can directly regulate gene expression at the transcriptional level, while circRNAs can switch off this regulation by sponging miRNAs. Dynamic changes in the free content of miRNAs and circRNAs can lead to the expression of target genes being turned on or off (up or down), while these changes in the interrelationship usually represent the occurrence or progression of diseases [20].

In recent PD studies, circFTO and miR-187-3p have been shown to be associated with disease progression. Compared to normal subjects, we found that the expression level of circFTO was significantly upregulated in the SN of patients with PD, indicating that circFTO can sponge miR-187-3p, driving dysregulation of the mechanism by which miR-187-3p regulates EEF2, thereby accelerating the progression of PD. In our study, we found that disrupted regulatory pathways of circFTO, miR-187-3p, and EEF2 exacerbated oxidative stress and inflammatory factors in brain, in a manner possibly related to the progression of PD caused by chronic inflammation. As such, the circulating levels of circFTO can be used as a biomarker for the early diagnosis of PD to determine the risk level and disease severity of the population because normal or even low levels of circ-FTO content can reduce the adsorption of miR-187-3p, thereby inhibiting the potential inflammatory activation of EEF2/EEF2K. The results obtained in experimental animal brain tissue samples also showed that targeting circFTO could effectively improve PD symptoms, especially somatization symptoms, indicating that it is a potential therapy for PD, functioning reduce neuronal apoptosis and regulate oxidative stress and inflammatory factors in the brain microenvironment. However, the action of miR-187-3p may be a double-edged sword, as this miRNA can bind to the coding sequence of Seipin, a protein which can hinder neuronal apoptosis and inhibit the transcription of Seipin protein, resulting in increased neuronal apoptosis in brain injury [21]. In the present study, we found that miR-187-3p is an important factor that inhibits the high level of EEF2/EEF2K; however, excessive miR-187-3p can block the normal protein synthesis function, which is not suitable for normal life activities. Therefore, it is important to explore the beneficial content of miR-187-3p in subsequent studies.

Notably, previous studies have shown that the inhibition of EEF2 phosphorylation by EEF2K can reduce AS neurotoxicity and achieve therapeutic effects [15]. Therefore, neurons with low expression of miR-187-3p and/or high expression of EEF2 can regulate the expression disorders through extracellular vesicle infusion to increase the levels of miR-187-3p, thereby reducing the neurotoxicity of AS in the SN or striatum. Kameshima et al. found that knocking down EEF2 and reducing EEF2K activity significantly inhibited low-energy environment-induced neuronal apoptosis and autophagy [22]miR-187-3p inhibitor attenuates cerebral ischemia/reperfusion injury by regulating Seipin-mediated autophagic flux, as high levels of EEF2 may represent worsening symptoms of neurodegenerative diseases. Our findings demonstrate a regulatory relationship between miR-187-3p and EEF2, which could help to hinder excessive neuronal apoptosis in early PD processes, as miR-187-3p interferes with the translation of EEF2. Although our study demonstrated the contribution of circFTO and miR-187-3p in PD, it did not prove the contribution of these factors in practical applications, as ncRNAs have multiple downstream targets. In future, we aim to combine slow-release materials to modify circ-FTO and miR-187-3p to explore the efficacy and safety of targeted controlled-release agents for the treatment of PD.

## 5 Conclusion

cricFTO can target miR-187-3p, and miR-187-3p can target EEF2, and their expression levels can predict the risk of PD progression. Targeting circFTO can reduce the levels of brain risk factors including inflammatory factors TNF-α and IL-1β, as well as SOD and MDA, which can enable PD to achieve a certain therapeutic effect.

## CRediT authorship contribution statement

**Jiahao Feng:** Writing – original draft, Supervision, Software, Project administration, Formal analysis. **Jin Zhao:** Methodology, Data curation. **Yong Kuang:** Validation, Formal analysis, Data curation. **Yuheng Zhou:** Methodology, Investigation, Formal analysis. **Ziheng Ye:** Software, Resources. **Yutong He:** Software, Resources. **Li Zhang:** Visualization, Validation, Conceptualization. **Tingying Zhang:** Validation, Software. **Qingqing Zhu:** Validation, Supervision, Formal analysis, Data curation. **Shumin Cheng:** Writing – original draft, Validation, Supervision. **Taoli Liu:** Writing – original draft, Supervision, Funding acquisition.

## Data availability

Publicly available datasets were analyzed in this study. This data can be found here: https://www.ncbinlmnihgov/geo/query, http://www.biomedical-web.com/circmine/, https://starbase.sysu.edu.cn/.

## Funding

This manuscript was funded by 10.13039/501100010883Traditional Chinese Medicine Bureau of Guangdong Province (no.20231077), 10.13039/501100021171Basic and Applied Basic Research Foundation of Guangdong Province (no.2022A1515220096), 10.13039/501100017607Shenzhen Fundamental Research Program (no. JCYJ20230807110417036), the Open Fund of Shenzhen Key Laboratory of Chinese Medicine Active Substance Screening and Translational Research (No.ZDSYS20220606100801003).

## Declaration of competing interest

The authors declare the following financial interests/personal relationships which may be considered as potential competing interests:Taoli Liu reports financial support was provided by 10.13039/501100010883Traditional Chinese Medicine Bureau of Guangdong Province, China; 10.13039/501100021171Basic and Applied Basic Research Foundation of Guangdong Province, China; 10.13039/501100017607Shenzhen Fundamental Research Program, China; the Open Fund of Shenzhen Key Laboratory of Chinese Medicine Active Substance Screening and Translational Research, China. If there are other authors, they declare that they have no known competing financial interests or personal relationships that could have appeared to influence the work reported in this paper.
