# Supplementary material for: Hsa_Circ_0105596/FTO inhibits progression of Parkinson's disease by sponging miR-187-3p and regulating eEF2
Source: Heliyon. 2024 Nov 2;10(23):e39830. doi: 10.1016/j.heliyon.2024.e39830 (PMC11626004; doi:10.1016/j.heliyon.2024.e39830)
Supplement: Multimedia component 1 [file mmc1.docx]

Supplementary Table 1 Trends of Degs in Striatum of PD

| Degs expression profile | Gene | number |
| --- | --- | --- |
| LogFC>0.3, p.adj < 0.05 | LRP1, PDLIM5, TIMP3, SMARCA1, NF2, MDFIC, ADRB1, RASSF8, PTPRF, PARVA, MAPKAP1, RBMS3, HIPK1, VEGFA, CPT1A, MYL9, FGF2, EHD2, MAZ, GALNT2, MINK1, RRAS2, RAMP2, ZEB1, TBC1D1, SYT5, PODXL, QRICH1, PRELP, GAS2L1, NR2F2, ZRSR2, MEN1, HMG20B, TGFB1I1, ITIH5, FADS2, NOTCH3, CLIC1, SOD3, ARHGEF10L, MCC, CACNB3, ITGA7, WWTR1, TRAF3, VAMP2, KDELR1, CRTC1, GABARAP, ATP10D, YKT6, ZNF710, PIK3C2A, TCF7L1, CCDC86, CTBP2, FLNA, FLT1, ASPH, CHRNB2, CLU, TRIM14, HIF3A, PANX1, GPATCH8, TRPM3, ATP10A, STX18, IGF1R, GPR27, LSM14B, ZC3H13, NHLRC2, GRIA1, COL4A2, CLSTN1, IRF2, PXN, RYR3, GUCY1A2, MLC1, APC, MTO1, MEST, PC, ARHGEF15, GPD2, ADORA1, LTB, PDLIM2, KIF13A, SOS1, LDB1, CACNA1B, ARHGAP5, THRB, ERBB2, ASL, CAPN2, KLF3, SLC12A6, HIC1, CCDC93, RAB11B, CIZ1, DGKH, RHBG, USP12, SLC6A11, SLC39A4, SMG6, CAMTA2, INCENP, TMEM53, BCR, EZH1, SH3GLB1, SERPINB9, SLC16A1, FBXW2, FOSL1, COPZ2, RHOBTB2, ARHGDIG, CNKSR2, ZNF623, KTN1, RAP2B, ATRX, AFF1, IGFBP4, TNS1, FEM1B, NRF1, PHKA1, OGFRL1, NRG2, MBD2, GPR135, SLC30A10, ERC1, RFK, RAD52, GNG12, SAMD4A, CCNG2, HIPK3, NF1, ABHD11, PGPEP1, BCAS3, SCN3A, TNR, NPEPPS, TNKS2, RPS15, IQCE, GLUD2, SKI, NCR1, CSDE1, ZC3H7B, PLA2G12A, TEAD3, LBH, POMC, PYY, FAHD2A, AKT3, TNKS, METTL8, CCDC134, CACNA2D1, BANF1, ALS2CL, GRIA2, NYX, ZDHHC4, ENTPD6, VPS13C, ANXA2, RNF146, PTPRS, JUN, RAB3A, ZNF747, PDZRN3, HMOX2, LRP6, PPP2R5B, SART3, TTBK2, PITX1, YWHAE, BACH1, ERBB4, ECM1, PCTP, SOCS6, FGFR1, HDLBP, GLUL, ONECUT1, DOCK4, RNF208, ADM2, TGFB2, PTPRCAP, CRYAA, ITGB8, AGER, STK17B, CARD10, PDK1, PAFAH1B2, DENND4A, DGKZ, AGRP, REST, CCNL1, CDC73, MAN1A1, FRY, CAPN10 | 225 |
| LogFC<-0.3, p.adj < 0.05 | KCNK1, INSIG1, ZNF226, PCSK7, UBE4B, PRRG1, NDE1, RFXAP, STMN4, TF, ATG3, TGFA, MOSPD2, SH3GL3, COIL, IPO13, TRIM24, APIP, PCCB, HMGCS1, TRIAP1, RAP1GDS1, SEMA4D, WWP1, PLA2G4C, CHD1, OSTF1, SLC26A2, MTF2, ANKZF1, SLC30A5, MAP6D1, ST3GAL5, TSPAN8, SLC35A5, TMEM38B, THBS2, GCA, GRM3, MAG, ENPP4, GUSB, HSD11B1, GPR37, NGLY1, JAM3, PMP22, RPP40, LRRC8D, SMARCE1, PIGK, PAQR4, MTMR6, SCARB2, SPOCK1, TNFRSF21, DLC1, NSDHL, PLCL2, TNFAIP6, SPATA5L1, SCG5, GALC, PTPRE, PAPSS1, GOLGA5, MSH6, EXOSC7, LIN7B, LARP6, TXNDC9, ZNF451, LANCL1, AGK, EIF2B3, LBR, NASP, ATP1B1, SKAP2, AGA, GLA, ZNF365, LITAF, MMD, PGRMC2, MKRN1, FH, CD46, PPP1CB, SLC19A2, PCNA, SNAP25, ETV5, RNF103, RABGGTB, NUP107, RBM15, CXADR, EPB41L3, OSBPL1A, RNF138, NINJ2, ENOPH1, DYSF, CPD, PPRC1, POLG2, ANAPC5, PPP1R16B, UGCG, ATP2C1, TOMM20, THAP10, PMP2, ACAT2, OXR1, TMEM87A, CLDND1, CLCN3, CHMP2B, STAMBP, SLC30A9, LAMP2, SERPINI1, VBP1, BUB3, RAB40B, PIGN, RALGDS, RPS6KB1, PIK3R4, RCOR3, XPC, ACTR6, ALG5, SCD, STAT1, ESF1, LIPA, ABCC5, ALCAM, PHTF2, NDRG1, RYBP, FOXN2, RAB22A, COL16A1, INPP1, LLGL1, GOLGA7, ZFAND1, DCK, ZNF529, BCHE, CUTC, ARIH2, SREBF2, FIS1, OLIG2, GTF2H5, KLF13, LDOC1, HDAC2, HMBS, SSBP1, MDH1, ANK3, PIGA, KCTD5, VRK1, IBTK, QDPR, REEP5, MRPL9, NKTR, PSMA3, DDHD2, RPA2, GSK3B, CAMTA1, VPS4B, AP4E1, HLTF, HMGCR, NFU1, NUDT1, PTS, CELSR2, CRYZL1, SYNJ1, PELI1, ATG4A, PGRMC1, SLC11A2, GM2A, GNL2, NDUFV1, GAMT, DHX30, COL4A5, NR4A2, MCCC1, PPP2R2A, FGF9, TTF2, PRKCZ, NCLN, TIAL1, TULP4, EXOSC8, COPS3, CDC23, UBL3, PSMD14, RGS12, HSD17B4, ABCA12, TRIM36, RTN4, CHKA, RABEP1, NIF3L1, PPIG, SRRM1, SACM1L, GOLT1B, SLC6A15, RRM1, PFDN4, KNTC1, RNF13, THUMPD2, SFRP1, KIFAP3, SH3YL1, SLC12A9, ATP6V1E1, GNE, ZDHHC14, MRPL19, PHLDB1, TAGLN3, ATP1A1, GCNT4, SNCA, MTMR9, ACTR2, PHLDA3, PIGP, GOT1, APAF1, ANKMY2, PPP2R2B, ARPC5, EPM2AIP1, POLR2B, ARHGEF2, SCAMP1, CD9, HBB, SEC22B, ARHGEF7, RELN, TALDO1, ITGB1BP1, TDG, TRAPPC2L, FEZ1, MYNN, TRPC1, LAPTM4B, VAMP8, RPN1, NSUN6, GLTP, CCNG1, PCMT1, ITPA, DCTN6, RNF6, RTN3, ZNF330, USP14, PEG3, DHRS1, SAP18, IMMT, SNX4, MDC1, SEMA6A, FAM3A, PDCD10, AK2, APLP1, CIRBP, NLGN1, PJA2, EFHD1, SEH1L, RFC5, SCCPDH, ELF2, DERA, SRPK2, COL9A2, NUP133, FNBP1, METTL5, SH2D1A, MYLK, KLHL4, ASTE1, SCG2, TRIM13, THYN1, UCHL3, ATF3, BEST1, SLC25A14, GLCE, PREPL, SLC35B1, YIPF4, PAF1, EBAG9, CSTF3, ERCC5, TBCD, CTSO, TSPAN5, SLC36A1, ALPP, RIOK3, CLCA4, PPP2R5E, CRIPT, PFKFB4, PBLD, LEMD3, ADIPOR2, KCNJ2, ACOT7, BBX, ZNF10, HSPA2, MPHOSPH10, MAPK8IP3, AMD1, CNTNAP2, COPS7B, LRRC1, TCFL5, STK3, RDH11, PREP, MBD4, ADAM15, NANS, ATP6V1H, SCAP, MAPK6, NUDT15, ALDH1A1, OSBPL9, ZMAT4, CD55, METAP1, PPA1, RAB9A, CMAS, RALA, CAMKK2, ABCE1, PSMC2, EVI2A, SUCLG1, PCNT, SLC4A8, ARMC1, CNTN2, LSS, PKP4, MVP, TMEM144, MUTYH, TLN2, TJAP1, ASNS, CDK5RAP1, PEPD, TM2D3, CUL2, ASPHD1, EEF1E1, GMFG, CPOX, MLLT11, ARL1, SCNN1D, KLK6, AP1B1, POFUT2, ARL8B, MRPL48, SUB1, FAM20B, CPEB1, BECN1, GPD1L, SPHK2, KLF9, DYNC1LI2, RPL15, FRAT2, ARFIP1, ACSL4, CDC7, IARS2, ANKRD46, UTP6, THOC1, RSRC2, INTS8, GALNT3, SMAP1, KCNK12, DNAJB14, NOL8, CCDC25, DFFB, ABHD8, MRPL17, DYNLT3, USP33, IGF2BP2, COX6C, EML2, POLB, ADRM1, ALDH3A2, CRBN, MFSD1, DUT, GLG1, MATN2, PSRC1, COPS5, DHPS, NDEL1, ELMO1, STMN1, SCN1A, ZNF212, LACTB2, GLS, DUSP26, PRNP, CCNE2, TOPBP1, HSBP1, EFNA1, UTS2, PPP2R3C, PEX3, ETFB, GTF2A2, AK5, YARS2, RAI14, SCAMP3, FNTA, SGCE, NCOA4, CDK5RAP2, TFAM, SH3GL2, YTHDC1, VAMP3, SESN1, MYOT, GAS7, DNAH17, SMC4, BASP1, RNF34, TAF7, HSPBAP1, SCAND1, ATP2A2, OSBPL7, STEAP1, KCNAB1, NSL1, POLA2, FIBP, ITGB1, WDR12, COX5A, CALU, MRPL3, ZDHHC17, MAP7, CREG1, ATP6V1D, ZNF536, RAP2A, SYNJ2, ZNF180, RASSF2, TGDS, ARL6IP5, ABHD10, DYNC1I2, MAP2K1, DNAJA3, PELP1, ZNF34, PFN2, DDX1, SIAH1, MED4, RASGRP3, CBFB, SNAPC4, KLHL2, UNC50, PLEKHA1, MRPL15, LHPP, NECAP1, CCPG1, GDI2, ZNHIT3, GPS1, DPYSL4, NAALAD2, TRMT11, TIMP2, COPS8, OPLAH, SLC35A1, CYB5A, TBK1, PAQR3, CAMK2N1, NEK3, TFPT, PSMB7, AGPAT4, LY86, MRPL46, POLR3C, BPHL, EIF2B2, ZNF614, DYNC1I1, LZTFL1, PDE1A, OLR1, STK39, NAP1L3, RBBP9, RFC4, PSMA5, KATNB1, REEP1, SLC44A1, SPINT2, RAPGEF3, TMEM59, MFSD11, TM7SF2, CTNNA1, EXOC1, OAT, FCER1G, ASXL2, MAP1B, ASCC1, NOL11, RBPJ, RDX, SRP54, DPYSL2, FASN, SGPP1, WDR59, PAK2, CTTN, ZNF706, PDIA4, TUBA1A, COX7A1, CDC20, CCT8, CDK5R1, ATP2B2, IFIT2, NSF, INTS6, PRMT7, PTGS1, MCF2, GDPD5, KLHL3, ANGEL2, C1QA, PTPN11, KLHL7, MTRF1, RPS24, SNW1, SNAP91, YWHAQ, FEZ2, CCND1, ARMCX3, CDK7, SMARCA4, INSIG2, UGDH, INPP4B, OSGEP, GYPC, RAB5A, COL11A1, TMEM186, PDHB, NUDT9, ZNF562, SV2A, OSBP2, ENOSF1, NR1H2, ZSWIM1, NDUFB7, MIS12, ALDH7A1, RAPGEF6, THY1, MARCKSL1, NDUFS7, MYOM2, ITPR1, RAP1GAP, PROX1, SREBF1, DRAP1, STAT2, CTPS2, CCDC59, TMBIM4, UBE2E1, ASPN, CXCR4, BCAP29, SIL1, RIOK2, AKR1A1, CAPN3, EXTL2, FAM8A1, TTC31, TNFRSF14, SERTAD3, DLG2, PRKCQ, FAM98A, PCCA, RAB11A, AIP, CUEDC1, ENO3, ST3GAL4, RBX1, NSMCE4A, MTX2, MYCBP2, COQ3, LRRC49, CHCHD7, CYCS, RTN1, SCRIB, CEPT1, RNASEH1, SNX10, CUL5, DDX27, SLC22A17, AP2S1, MTMR4, OVGP1, POP4, CUTA, MRPS17, NPC1, MAP1S, ATP6V1B2, BAZ2B, MKRN2, AZIN1, BTBD3, NCKAP1L, ZNF217, ASNSD1, SBF1, MRPS22, SRPRB, ACSL1, PCID2, TBCE, ELP3, FUCA1, CENPB, NIPA2, PARP1, POLR1C, NTHL1, MAP3K7, SEC11A, ING1, DHX29, SS18L2, SAMM50, DMRT1, UBE2V2, ZNF23, ZFAND5, MYL6B, TTC1, SULT4A1, LMBRD1, CCT6B, EDC4, MTRR, PDGFRL, CEBPZ, PIGF, AASDHPPT, MCTS1, EIF2B4, ATP1B3, SH3GLB2, MTMR14, TMEM30A, GOLPH3, PWP1, PTK2, PSMA2, RPS6KA5, WAS, SLIT2, DIAPH2, ATP6V0E2, COPS4, SPRR1A, GALK2, HSF2, LAMP1, PDE6B, SLC25A4, RAP1B, SV2B, MCM7, HEXB, ZNF189, CBX3, ATP6V1A, GLRX, TMED3, SLC31A2, GLO1, CKLF, TBXAS1, SEC61G, TSSC4, FBXO5, FASTKD3, CHRNB3, TRIM37, RBP1, DHRS9, NBN, TST, NDUFB2, PPCS, PPP3CB, MYT1, TBCB, SS18L1, ARMCX2, FAIM, HDDC2, DUSP12, XPNPEP1, RASSF7, HSPA14, ACLY, DNM3, HINT1, VDAC3, PPP2CA, RB1CC1, LEPROTL1, COMMD3, ZNF428, DHRS7, CDC40, GUK1, ARCN1, CRYL1, GBE1, RNASE1, NPAS3, GTF3C5, MCM3AP, NDUFA2, NOC3L, IL21R, NDUFAF1, RYR2, HAGH, APOO, DNAJA2, GOT2, RABEPK, KLHDC2, PAK1IP1, NDUFS4, SYT11, KCNQ2, RAB33A, EEF1A2, USP1, EPN1, TMEM70, BTG3, PRKAR1A, PLAG1, AIF1, VPS41, PDHA1, VPS37C, SYPL1, PARP6, KLHL5, CHUK, UBE2N, CDC25B, DENR, PSMB5, TBC1D22B, PPP2R1A, DAZAP2, SLC9A1, DNASE2, VEZF1, EML1, LCMT1, SAR1B, STIL, RUFY3, SLC2A5, TSPAN13, STK38, TTF1, BIN1, PSMD4, SH3PXD2A, KPNA6, HEY1, DYNC1H1, CSPG5, CSRP1, COX7A2, ART3, SOX21, MOAP1, ADSL, HSD17B6, ARL2BP, TRIM23, PAQR6, IDH3B, TACC2, DEK, PHYH, RAE1, PSIP1, PIM1, ZNF415, GGPS1, PFKFB2, PUS7, YWHAH, PSAT1, DIXDC1, BTBD2, EIF1B, DYNLT1, TIMM17B, KIF1B, CCT2, RANGAP1, SF3A3, TIMM9, WBP2, SRRM2, HSD17B12, ZSCAN16, AP1S2, FEM1C, RNF14, GP1BA, POLRMT, ZNF235, PRPS1, NKX2-2, SLC37A4, KIF14, CAP2, NDUFS3, AVPI1, KHDRBS3, NRIP3, IQCK, PRDM4, GTF2B, CD47, PPBP, XRCC5, TKT, REV1, SPCS1, CYP2R1, CBR4, ELOVL4, MTMR11, SLC20A1, MRPS31, HLA-DMA, PIGB, TRAM1, RFX5, RPIA, AP3M2, KIT, SERPINA1, AHCY, METAP2, IFI6, GAPVD1, NRXN3, TERF2IP, RRAGD, DNAJC12, LTA4H, NDUFB3, FPR1, ALAD, SLITRK3, NUAK1, CHGB, LPXN, DCLRE1C, NCSTN, UBQLN2, RECQL, PSMB4, RNF41, PSMB3, IVNS1ABP, SUCLG2, GNAO1, CGGBP1, CALM1, TSEN2, RPS14, YWHAB, MOXD1, GABARAPL1, PITPNM1, DNAJB9, WASL, SETD3, CSE1L, MPHOSPH6, GRPEL1, KIF1A, FLNC, MPL, STMN2, BIRC3, PSMC3, RBM5, MRPL22, ACACA, COMT, PSMA4, TM9SF1, PRPSAP2, CAB39, ZNF223, DNAJB2, PRC1, SNRPB, ACP1, AKAP7, MAN2A1, DGUOK, STK4, FKBP1A, LY6E, SLC35E3, ACAT1, RRAGA, ENPP2, GHITM, OGT, OSBP, CEP135, KLHDC3, FSCN1, IFNGR1, IFI44, DNAJC7, RBM17, MOS, COBL, KBTBD2, BAMBI, UPF3B, MAP1LC3B, PLA2G4A, CDKN2C, HADH, MAT2B, DHX16, PSME4, ARID4A, LDB2, FXR1, BRD2, PPFIBP2, TIPARP, SLA, MAN2B2, TAF15, RGL1, PRPSAP1, PCDH9, CTH, SLC25A12, GNL3L, NME7, PIN1, INTS12, NDUFS6, PEX11B, DHCR7, PTDSS1, BCLAF1, DHX15, ATP6AP2, CBX7, UROS, KIF13B, MRPS10, FBXL2, BICD2, CRY1, UBL5, HERC2, IFT74, ACBD3, PPA2, SCPEP1, NAP1L2, ABHD6, AGPAT5, HPRT1, STX16, IFIT1, DGKQ, ESD, ALG8, RPS6KA2, CYB5R2, DCTN3, ENC1, PDSS2, ATOX1, CAP1, NDN, LYRM1, ITGB2, SCAMP5, TTC19, MTIF2, RNF7, TIPRL, GGH, NCOA6, DNMT1, PSMB10, PCBP4, EPB41L1, GAB2, SMPDL3A, POLR2I, MYOM1, ABCA8, POLD2, ERBB3, RBM22, TAP1, LAGE3, ARPC5L, MKKS, ALOX5AP, GRIN1, PNMA2, DEGS1, TMCO1, RSL1D1, TM2D1, CYFIP2, MRPL23, ENO2, MYLIP, POLE, ACTR1A, GLMN, ZCWPW1, XYLT2, TUBA1C, EML3, PPP2R3A, AMPH, RPL11, THOC7, WBP4, MRPS28, CAPZA2, RPL19, RAB11FIP3, CRISPLD2, KRCC1, HERC1, PLD3, MRPL40, SOX10, HIBCH, CLIP2, IMPA1, MORF4L2, DNAJA1, JUP, SNX27, YPEL5, HIC2, RRAGB, ETFA, NIT2, SUPT5H, IL18, NDRG3, SIVA1, CCT6A, RPL31, HLA-DMB, TXLNA, ATP6V0B, YIF1A, NPTX2, ASF1A, SST, PFDN5, ITPKA, TSNAX, GIMAP4, PAIP1, CDH19, AHR, SDAD1, DUSP3, GRSF1, STXBP3, TCF25, DCP2, MARCKS, COX7B, PSMB1, TMEM97, ADARB1, TUBA1B, CNBP, DSTN, NDUFA13, POMGNT1, ARHGEF3, CGRRF1, PTOV1, CLCN7, MPPE1, PRKRA, TNRC6B, RPS11, RPL22, TSR2, STK25, SERPINE2, SMAD5, ENY2, OLFM1, NISCH, DSE, FN3KRP, MRPS33, CDKL3, TSPYL1, RCN2, RPUSD2, ANP32B, DDX18, CA2, GALNT11, PTRH2, DYRK4, DOCK10, NPDC1, YY1AP1, FBXO21, TXNIP, GADD45A, EIF4H, ALOX5, IFRD1, SSX2IP, PSAP, NDUFA8, TMEM158, TACC1, RAB3GAP2, AKAP11, SOD1, GORASP2, TSPYL5, MGST3, POLR2G, RIT2, CDH9, FBXO11, TMEM9B, NDUFA4, CUEDC2, JUND, RIMBP2, PBX3, RPS27L, RALGPS1, BZW2, METTL1, UGP2, FLOT2, SAT1, ACRV1, PAICS, GFM1, HTRA1, SPN, SNX16, SF3B2, CLIP3, CNN3, DECR1, APOE, TEX2, VAMP1, UFM1, POLR3B, CCNI, EHBP1, CAMLG, COMMD9, LCP1, PMPCA, RPA3, CKB, RAB26, SCFD1, DKK3, E4F1, ANXA7, C1GALT1C1, RGS10, DPM1, HERC5, DUSP14, SLC17A5, GPM6B, SLC6A1, CHN1, MED6, MYO10, RGS17, CNDP2, ZNF143, PSMD10, HCFC1R1, SPP1, LYN, SASH1, NELL2, CDC37L1, PIAS4, COPS7A, RNF113A, RNMT, SDHC, NPC2, CASP3, DACH1, DCTN2, ITGAE, DBT, PMM1, RARA, GAD1, CCDC106, PEX5, ADCK2, NDUFB5, RHOT1, GUCA1A, NOL7, PSMA1, CRELD2, LXN, CD14, TRIM33, TOMM40, RIC8B, SEMA3B, TMEM14A, PTBP2, NEFL, PITPNB, CORO1A, HIRIP3, ZNF324, ROBO3, UBE2L3, ALG13, SNRPD2, UCHL1, LONP1, POLG, GSTO1, FAM50A, DPH2, LY96, HOXB6, PNMA3, WDR47, MRFAP1L1, CCL7, ZSCAN18, TSPAN9, ADD1, CAT, BLOC1S1, ZNF721, NDUFAB1, COX4I1, E2F3, PPT1, PFDN1, SIPA1L1, TRIM2, RUVBL2, NDUFB6, FOXJ2, ATP6AP1, PPARGC1A, SERTAD2, TBCA, MNDA, URM1, DLGAP2, MDM1, ZNF544, GFOD2, EIF4B, YWHAZ, AQP1, TCEAL4, GTPBP4, DIRAS2, ULK1, GCLC, PCBP1, PRMT1, UIMC1, VDAC1, SSR4, ZNF22, ANKS1B, IFIH1, COX5B, ZBTB48, RSBN1, OPTN, ATG12, TRIM9, CD86, BAIAP2, RNASET2, RASA1, ECHS1, PSMC5, INA, ACTR10, KCNF1, CLDN14, CRKL, TBPL1, CLEC2D, SUMO3, GPR162, EIF5A, NFKBIA, CACYBP, TRAK2, MAPRE1, UROD, ZNF93, APBA3, EEF2, EVL, EDF1, HOOK1, TBRG4, MAGED1, S100A13, VHL, RNF128, PAK6, PRR14, STARD7, ZNF14, UPP1, MRPL18, DLL3, TFG, ALG6, RPS23, GNG7, UBE2I, ACTL6A, CDC16, BUD31, POLR2F, COX6B1, SERPINB6, ARHGDIB, ADAMTS1, NFKB1, TFRC, TRAPPC2, TTC4, MYL6, POGK, SLC9A5, CPEB3, SCARB1, ACYP1, IL11RA, BRD9, DDX17, ATIC, PCNP, LSM7, ADM, PIK3R2, IQCG, SLC2A3, TOP2B, RGS4, CS, CA14, NEFH, PLEKHA5, WDR19, TNK2, IMP3, CETN2, WDR6, PRDX4, ASCC2, ATG10, SLC27A6, MGAT2, ARPC2, RPS15A, KRT10, GLRB, ARHGEF18, SCN2A, G6PC3, ZNF467, XAB2, AFF3, FGF13, TPD52L2, DYNC2LI1, FAIM2, CASP8AP2, LGALS1, RPL36, MXRA7, NMNAT2, TAX1BP1, CSF1R, CD74, CDKN1A, TYK2, MRPL33, POLE3, NDUFB8, CPS1, RHEB, LSM3, LARP1, PIR, CYBA, SRGN, CA10, COMMD8, SLC9A6, MPPED2, FAAH, NOL6, CNR1, CACNG3, VPS28, NDFIP1, CDO1, LIMK1, TRABD, LMO2, RGS20, CBX6, GAP43, CYP2J2, FCGR2A, NKRF, PDZD2, VNN2, EVI5, CRYZ, PGM5, LMO4, UGT8, TMSB10, FGF12, RHOB, BSN, VPS35, RPL9, ESRRG, CLK1, VSNL1, GTF3A, ABCF1, MRPL24, SNX3, ARF5, ZMPSTE24, NPTX1, LZTR1, AMZ2, TNPO2, RIMS3, ATP6V1C1, ZMIZ1, CELSR3, CDH10, EHD1, YY1, MYH10, POLR2A, NEDD4L, POLR1D, BET1L, SCRG1, LRRC23, SUZ12, CLTB, ARF4, NDP, GTF2F2, PHGDH, EPHB6, DEXI, PLEKHB1, SUOX, DBI, TM4SF1, COPB1, ANGPTL4, LPL, TULP2, RBM14, UQCRC1, REXO2, TMEM160, PPOX, MSRB2, CXXC1, EFHD2, TCEA2, NME1, PPFIA3, EIF2AK2, SIDT2, UBA52, TDRD1, ADCY3, CNTNAP1, SYF2, FTH1, HDAC1, XPO1, NAGPA, EP400, OPCML, HOXC13, SCG3, ALDH9A1, MAP4K5, VDAC2, MAPK3, NUDC, LSM2, TOB1, PHF11, RPS6, G3BP1, BCKDK, EEF1A1, LDHA, OR2B2, GTF2E2, GPR137, TSG101, GNG3, RASAL1, ADI1, LHX2, MAP1A, NUDT11, UBQLN4, KBTBD11, CFI, USP49, SLC25A22, AQP9, TAAR2, DIAPH1, UBE2L6, AKR7A2, CIDEA, BCAP31, NUP37, RPS6KA4, NPTN, NBEA, TRMT1, BLCAP, CRELD1, TYROBP, CCDC28A, KPNA4, TSC22D4, CDK5RAP3, UBE2D3, STK24, FBXW7, OSBPL2, IGFBP7, CKAP5, RHBDF2, ABR, CD44, ICA1, DPP6, ALDOA, LYPLA1, NME5, MORF4L1, KIAA0513, CHMP5, RPL30, CYB561, TIMM10, RING1, MAP4K2, RAB28, PFKP, PUS1, ANAPC13, LSM5, PRDX1, ZNF613, TPM3, CHGA, LAMA5, ECHDC2, EFNB3, HAMP, MYT1L, ZNF573, C1QB, ZNF83, SLC25A44, BNIP3, CACNA2D3, TCEAL2, SMARCD3, EDN1, NCDN, TAF1C, NRXN2, ID3, RPL36AL, UQCRB, NPY, CX3CL1, HPCAL4, FAU, DLG4, RPS16, MZF1, TUBB2B, SPOCK2, ASCL1, RAB5B, RGS7, DNAJC8, NCKAP1, HSPE1, SLC24A3, OAZ1, CDK5, UQCRQ, CCDC6, LRRN3, ADAM23, GRIN2C, GAD2, PARL, PNPO, EWSR1, RENBP, NUP62, IL4R, AKR1C3, RGS11, INPP5A, GNS, SYP, MTCH1, BCL11B, COQ10B, GABBR2, STIP1, RPS29, MRPL13, RPS19, NDUFA3, MRPS18C, USP5, NRGN | 1785 |

Supplementary Table 2 Trends of Degs in Substantia Nigra of PD

| Degs expression profile | Gene | number |
| --- | --- | --- |
| LogFC>0.3, p.adj < 0.05 | PCK1, PTPN2, MYCN, HSPA1L, CLIC2, CASP4, IL7R, MYOT, BRCA1, TLR5, ASCL2, BCL2L11, KCNE4, TCL1B, CASP1, C21orf62, TRAF3IP3, RNASE3, SNAPC1, MICB, AGBL3, ITGAM, RAD52, APAF1, ADAMTS1, PLA1A, LY96, RELN, TAL1, FCGR2B, CDH1, LRRC32, MYO9B, LHX6, LILRA1, IL18R1, TLR7, SERTAD2, PELI2, PABPC3, CLEC2B, NIPBL, PYCARD, PEX6, S100A12, IRF7, SP110, DNMT3A, FCGR2A, CACNA1I, LRRC1, BIRC3, ZNF225, MICA, GLI2, P2RX1, KIF14, BDKRB2, CSTA, ZNF350, OR1D2, STARD13, CDK6, ETV1, CPM, NKX2-2, LILRB2, F11R, FZD9, FZD2, RNASE2, DCLRE1C, DGKD, MYO1D, SLC22A3, PDLIM1, MAK, NOX3, CR1, HIST1H1T, IL1RN, MGAM, PTPN22, HIST1H2AC, MTHFD2L, VSX1, CD3G, CDK2, USH2A, IL19, ROM1, KRT6A, CYP3A43, RPA4, MAF, TUBD1, TBR1, TEP1, MAP3K8, CBLC, TSPAN14, SLC39A8, EIF4EBP1, ABCC3, MMP13, PHF20, GCM1, EVI2B, CARHSP1, LHFPL2, CEBPD, VSIG4, TNFRSF11B, TRIM22, ZNF185, SEC14L4, CCL16, ZNF395, CPA3, KCNK7, YES1, GIMAP4, VIPR1, DSG1, SMR3A, LGALS9, TCF12, NOTCH1, GZMA, TEKT2, CEACAM5, VPS13C, HIST1H1C, TCF4, WNT7B, TEAD4, PRKX, NR2E1, TRAF1, CLDN14, KCNMB4, FOSL2, TEX12, EDN2, HCP5, HIST1H2BM, IFNAR2, APBB1IP, KRT12, ZNF442, FLT3LG, SLC27A3, TCIRG1, NFE2, HIST1H4I, FYCO1, TCF7, TMEM45A, CXorf21, PAX6, TLX2, MAN2A1, CLMN, SLCO4A1, DEFB126, TNIP2, TNFRSF14, GAS1, ADAMTS20, AXIN1, MSX1, HIST1H3A, MAGEB1, TYRO3, GPR65, PDGFRL, CXCL12, UBQLN3, ITGBL1, CYP24A1, ETV4, PLCXD1, SLC5A12, DPEP3, GNA15, SLC25A31, PTPRK, RIBC2, TSPAN1, MKLN1, CARD8, ST18, CFH, PALMD, PNMT, DUSP5, PRPS2, ALOX5, TRIM38, HERC5, KLHL5, MAP3K6, SVIL, TNFRSF10B, CEP55, CNTFR, CTDSP2, TMEM39A, CDH19, CNOT6, CLIC1, BTBD7, UCP3, CILP, LMO7, TRPC7, GLP1R, CLCA4, TBL1X, CCR5, FGL2, TXNIP, CDH16, SCN4A, SCEL, PLEK2, MORC1, KRTAP5-9, ACSL5, BCHE, NFKBIA, ANGPT1, NEDD9, RRBP1, SLC13A3, DIAPH2, TNFAIP6, RP2, THEG, TYRP1, MYOD1, CXCL9, DDO, HIST1H4A, FMO2, MAFF, RPL31, SSTR5, FOXF1, PSG5, PRB3, LENEP, LPXN, NEDD4, MYO5C, ADA, NAB2, IL6, EGFR, POLE, ZNF143, FCN3, ZC3HAV1, AZGP1, FGF23, SH3BP4, NPHP1, C1QB, CUBN, SLCO5A1, STIP1, BBS1, NPTX2, MID1IP1, DUSP10, LILRB1, CABP2, CFLAR, PSCA, NOTCH2NL, SIX3, ZNF302, IL4R, GBP1, HK2, OR2C1, H3F3A, PTTG1, BAZ1A, KLRB1, ATP4B, ZBTB20, TCL1A, EHF, ZIC3, OSM, CD209, MMRN1, BNIP2, ITPR3, SLC10A1, NANOG, OR2B2, KLHL4, CDYL, APBB2, KRTAP1-3, GZMH, MSMB, CACNG5, ERMAP, HNF4A, CLEC1A, HIGD1B, GFI1, SERPINI2, PAPPA2, GUCA1A, CNOT2, FOXC2, LIPF, TNNC2, HRC, PABPC1, CDKAL1, ICAM2, KLKB1, ZFP36, TUBAL3, PRRG4, SLC10A2, POU1F1, CASP6, SERTAD3, HGD, KCNJ5, HLA-DMA, NXT2, CLDN9, ZC3H7B, PTGER4, TEAD3, RAC2, A4GNT, GZMK, HAO2, TSEN2, SPG21, KIR2DS4, NGFR, DTYMK, FAS, LRP10, PLCE1 | 354 |
| LogFC<-0.3, p.adj < 0.05 | ANK1, RBM3, ALDH1A1, RIMBP2, AGTR1, ATP2A3, TUSC2, GRIA3, TPBG, KCNJ6, RET, OSBPL10, EN1, PIN1, TBC1D22A, LXN, SLC18A2, APBA1, ATP8A2, PTBP2, CCNC, CPLX2, ATP6V1G2, NRXN3, TUB, SYNGR3, SV2C, PCSK1, CBLN1, SCN3B, ASB6, CDH8, CMAS, NDFIP1, PSMD8, OGDHL, KIAA0319, SNCG, MECR, DDC, CNIH3, TBC1D9, C17orf75, MAGEL2, RIMS1, C12orf10, DCC, LBH, GALNT2, SULT4A1, PRKAR2B, RGS6, VAV3, DDX41, SLC25A4, AP2M1, PAK6, ARHGEF9, NR4A2, RGS4, CDK5, PSME3, RAB11A, OCRL, SV2B, SSTR1, KIF3A, DNM1L, TAGLN3, DMXL2, DDHD2, GPR176, NDUFA10, FBXL15, RTN2, ATP6V1H, UBE3C, LDB2, PLEKHA6, SCG2, CDC42, ATP6V0B, GPRC5A, CNTN6, GLS2, REEP1, DDX10, SNX10, ACOT7, UROS, CERK, MAP3K12, PRDX2, PCDH8, CRYZL1, TUBB3, STMN3, CLSTN3, SLC4A3, MID2, RNF41, BICD1, SPCS3, SYT1, REPS2, TSPYL5, TRAF3IP1, SEC23A, CADPS, INPP4A, KIF3C, SPINT2, STX18, PPFIA2, CLSTN2, TERF2, SMARCA4, BBS7, L1CAM, FBLN5, GSTA4, GSS, RCBTB1, NDUFA5, CDC37L1, AR, MYRIP, TRAK1, ARFGEF2, HPRT1, CEACAM21, MPI, NECAP1, PDK3, HYOU1, PTDSS1, DYNLT3, TMEM14B, RASA3, C1D, SCRN1, GABRB1, SGSH, KCNB1, ATP6V0C, RTF1, SLITRK5, TOM1L2, NRN1, MAGED1, PSEN2, DDA1, NELL2, ACP2, TSPYL2, PEX7, COPS7A, FABP7, GALT, NRIP3, GRIA1, ACTR10, DLD, TCEA2, BCL2L13, ATP13A2, NAPA, ZNF219, UBE2V2, FIBP, SNCB, ARHGDIA, PPP1R2, CLTCL1, ACTR3B, TM7SF2, RAB36, DHRS7B, RNF14, DENR, SLBP, NIT1, TH, UGP2, NUDT2, CCDC28A, PPOX, CACNA2D2, ELOVL6, NAT10, ATP6V0D1, TUBGCP5, BASP1, ELAVL2, NMNAT2, SLC23A2, FBXO9, TTC1, AP1S1, VDAC3, DNM1, DYNC1I1, YKT6, IRAK1, GNG3, SEPT6, SPA17, VAPB, KIFAP3, SLC6A3, SLC25A32, TRPC6, B4GALT6, CREB3, AFG3L2, SMYD5, SLC8A1, SUPT4H1, KIAA0895, ACAT1, CDS2, CIAPIN1, OPA1, GMPR2, AACS, DDX25, CCNA1, ZNF593, OSBP, PIP5K1B, INA, GAP43, FBXO3, ASL, UCHL1, EPHA5, CGREF1, CNTN1, GPR161, DLK1, STX1A, PPP2R1A, MFN1, MAP2, CHRNA6, MFN2, MAN1C1, IMMT, SLC38A1, RIT2, GFRA1, MED8, CAMK1G, GCH1, GLRX5, SLC41A3, PTPRU, SYNGR1, PDE4D, PPP2R2B, CYB561, AMPH, FBXW7, STUB1, FNDC4, DNPEP, WDR37, SLIT2, RAN, PRPF19, SCO2, TUBB, PSMA3, SCAMP5, SNX17, PITRM1, UBE2E1, FXR2, VPS39, TIMM13, ATP2B3, LSM5, DCTN2, MAPK8IP2, RPP25, CYFIP2, ICMT, LPIN2, CKB, EEA1, HRAS, ACTR1A, POP7, CORO2A, KLHL12, NDRG4, RIMS3, HCRTR2, NDUFV2, SLC9A6, MOAP1, USP12, NISCH, NAGPA, GABBR2, STS, FKBP11, SNRK, PRMT1, SRD5A1, GPRASP1, EVL, ABCC1, MCC, XK, ALAS1, C6orf106, NSF, ST6GALNAC5, CNKSR2, ADAM23, CX3CL1, FECH, AKAP12, SEPHS2, SMAP1, KCTD9, SUSD4, VPS52, SKIL, NDUFB5, CCK, BAP1, RHBDL1, FRY, DDX42, ATP6V1B2, EPB41L1, LMO3, NDUFA13, NRXN2, ELAVL4, PLD3, MCF2, RNF128, ZNF226, GABARAPL1, WDR7, SCN7A, LIN7B, MYCBP2, DNAJA2, CPNE6, MIF, TSPYL1, SUMO3, C16orf58, CLTB, PPP2R5B, C12orf49, NEDD4L, MADD, TM2D3, RANBP9, MYO5A, KPNA2, ARHGEF4, MAPKAP1, LRRC49, SCN2B, FBXL2, DRD2, FKBP9, LRFN3, NDUFA9, VBP1, SH3BGRL3, CACNB3, SPHK2, CYB5R1, CUL1, LMCD1, NEFH, BLCAP, ISOC2, GIPC1, TRIM36, IQSEC1, EPHA7, NETO2, CRBN, WSB2, FHOD3, AKT1, RBX1, CDK5R1, KCNQ3, PITPNA, PPP3CA, ATXN3, MAGI1, PIGZ, SLC20A1, TBC1D19, THY1, DDX1, LPO, CYP2R1, SNCA, DPYSL3, SERGEF, YWHAZ, ANK2, ABCA3, SCG3, GSTZ1, PPCS, TOMM20, PSMD2, TSNAX, AKAP6, DHRS7, ATP1A1, DCTN3, AKAP13, XPOT, PSMD10, MCFD2, KIAA0513, NSDHL, GPR173, PSMC5, ASB13, KPNA6, PDHX, RRAS2, CES2, CBX6, MTX2, GNB1, NME5, CDK7, ARPC5L, CNTNAP2, SYP, ACHE, NEDD8, CHL1, CHD5, NAPG, FDX1, ACTL6B, VAT1, TNFRSF21, CAP2, UBE2N, CDK5R2, GLO1, PQLC1, ARCN1, MRPL23, CD200, ASH2L, PSMD13, NFE2L1, ARL4C, BPHL, ATP6V1F, ACTR6, WDR45, WDR47, PTPN9, LRRN3, NOC4L, HABP4, CDC42BPB, HMGCR, GPX3, SFXN1, MPPED2, PAQR3, ERCC2, NUP93, SCPEP1, PRPS1, NOS1AP, ABCA5, BSN, EEF1E1, ABHD2, RHOQ, FGF12, SAR1A, PRKAR1B, PSMD7, HCFC1R1, NDEL1, SURF2, PCCB, DDX50, COPS5, HIRA, NRAS, PAK1, PSMC1, FGF13, CHML, BDNF, DNAJB9, LDOC1, INSIG2, RAB5A | 512 |

Supplementary Table 3 Trends of differential expression of circRNAs in PD

| Degs expression profile | Gene | number |
| --- | --- | --- |
| LogFC>0.3, FDR<0.05 | SHPRH, SOBP, SLC30A6, ATP5F1C, PACRGL, TSNAX-DISC1, DISC1, PPP1R13B, LCOR, CDYL2, UBR5, SASH1, FUT8, ZNF91, CFLAR, FAT3, LYPLAL1, KIAA1841, TMEM165, FNBP1, TERF2, ZNF609, ZNF124, FANCL, FGD4, ATXN10, TADA2A, FBXO9, BTBD7, FTO, ARHGAP32 | 31 |
| LogFC<-0.3, FDR<0.05 | KLHL24, ZNF292, ANK3, FAM135A, KATNAL2, NCOA2, AFF4, ZNF493, CTD-2561J22.3, GALNT13, ERCC6L2, EMB, AAGAB, BAZ1A, STXBP5L, KLHL1, FTO, SLC8A1, VIRMA, RAB3GAP1, TMEM135, FMN2 | 22 |

Supplementary Table 4 Trends of differential expression of miRNAs in PD (GSE110719)

| Degs expression profile | Gene | number |
| --- | --- | --- |
| In fibroblasts, LogFC>0.3, FDR<0.05 | hsa-miR-6075, hsa-miR-718, hsa-miR-5571-3p, hsa-miR-7108-5p, hsa-miR-6796-3p, hsa-miR-1181, hsa-miR-6794-3p, hsa-miR-3939, hsa-miR-663b, hsa-miR-569, hsa-miR-4745-3p, hsa-miR-5582-3p, hsa-miR-522-3p, hsa-miR-1301-5p, hsa-miR-4632-5p, hsa-miR-4467, hsa-miR-4426, hsa-miR-4665-3p, hsa-miR-2113, hsa-miR-2117, hsa-miR-1256, hsa-miR-1273d, hsa-miR-1914-5p, hsa-miR-642b-5p, hsa-miR-4501, hsa-miR-6724-5p, hsa-miR-7106-5p, hsa-miR-3160-3p, hsa-miR-8067, hsa-miR-512-5p, hsa-miR-3960, hsa-miR-519b-3p, hsa-miR-6743-3p, hsa-miR-1909-3p, hsa-miR-1292-3p, hsa-miR-3201, hsa-miR-6890-3p, hsa-miR-3657, hsa-miR-3672, hsa-miR-6088, hsa-miR-4747-3p, hsa-miR-3178, hsa-miR-5582-5p, hsa-miR-548ag, hsa-miR-4653-3p, hsa-miR-4329, hsa-miR-3196, hsa-miR-3195, hsa-miR-6729-5p, hsa-miR-519a-3p, hsa-miR-6756-3p, hsa-miR-4456, hsa-miR-6893-3p, hsa-miR-4514, hsa-miR-4532, hsa-miR-18b-3p, hsa-miR-619-5p, hsa-miR-604, hsa-miR-489-5p, hsa-miR-4454, hsa-miR-4281, hsa-miR-4436b-5p, hsa-miR-4726-5p, hsa-miR-517c-3p, hsa-miR-1298-5p, hsa-miR-4486, hsa-miR-5690, hsa-miR-4320, hsa-miR-489-3p, hsa-miR-4497, hsa-miR-8062, hsa-miR-4793-5p, hsa-miR-490-3p, hsa-miR-4485-3p, hsa-miR-302c-3p, hsa-miR-4665-5p, hsa-miR-3190-5p, hsa-miR-5700, hsa-miR-646, hsa-miR-1469, hsa-miR-3614-5p, hsa-miR-6775-5p, hsa-miR-4512, hsa-miR-95-5p, hsa-miR-6819-3p, hsa-miR-6872-5p, hsa-miR-4441, hsa-miR-302d-5p, hsa-miR-3659, hsa-miR-3973, hsa-miR-3920, hsa-miR-4707-3p, hsa-miR-6089, hsa-miR-4696, hsa-miR-1282, hsa-miR-4722-3p, hsa-miR-2277-3p, hsa-miR-4463, hsa-miR-1915-3p, hsa-miR-6789-5p, hsa-miR-1539, hsa-miR-635, hsa-miR-5787, hsa-miR-5004-5p, hsa-miR-6887-5p, hsa-miR-4646-3p, hsa-miR-5189-3p, hsa-miR-4634, hsa-miR-1236-3p, hsa-miR-7113-5p, hsa-miR-6816-5p, hsa-miR-1273g-5p, hsa-miR-20b-5p, hsa-miR-582-3p, hsa-miR-3181, hsa-miR-4787-3p, hsa-miR-4449, hsa-miR-1298-3p, hsa-miR-1972, hsa-miR-6735-5p, hsa-miR-1248, hsa-miR-372-3p, hsa-miR-3064-5p, hsa-miR-4764-5p, hsa-miR-516b-5p, hsa-miR-4787-5p, hsa-miR-4707-5p, hsa-miR-4516, hsa-miR-3621, hsa-miR-1237-5p, hsa-miR-663a, hsa-miR-4437, hsa-miR-6882-5p, hsa-miR-1908-3p, hsa-miR-1279, hsa-miR-6763-3p, hsa-miR-6869-5p, hsa-miR-3175, hsa-miR-3928-5p, hsa-miR-1537-5p, hsa-miR-2114-3p, hsa-miR-3678-5p, hsa-miR-5585-3p, hsa-miR-2114-5p, hsa-miR-4431, hsa-miR-4999-3p, hsa-miR-3943, hsa-miR-1273h-5p, hsa-miR-155-3p, hsa-miR-3618, hsa-miR-4669, hsa-miR-6873-5p, hsa-miR-6854-3p, hsa-miR-521, hsa-miR-4484, hsa-miR-5095, hsa-miR-4466, hsa-miR-95-3p, hsa-miR-3936, hsa-miR-4803, hsa-miR-6499-3p, hsa-miR-4472, hsa-miR-4664-3p, hsa-miR-183-5p, hsa-miR-4479, hsa-miR-4790-5p, hsa-miR-6781-5p, hsa-miR-6873-3p, hsa-miR-3155b, hsa-miR-3157-5p, hsa-miR-4445-3p, hsa-miR-6723-5p, hsa-miR-4258, hsa-miR-4417, hsa-miR-6802-3p, hsa-miR-5588-5p, hsa-miR-5193, hsa-miR-503-3p, hsa-miR-4788 | 179 |
| In fibroblasts, LogFC<-0.3, FDR<0.05 | hsa-miR-125a-3p, hsa-miR-574-5p, hsa-miR-629-5p, hsa-miR-1180-3p, hsa-miR-93-5p, hsa-let-7a-5p, hsa-miR-4760-3p, hsa-miR-132-3p, hsa-let-7e-3p, hsa-miR-2277-5p, hsa-miR-152-5p, hsa-miR-103b, hsa-miR-103a-3p, hsa-miR-192-5p, hsa-miR-30c-5p, hsa-miR-324-3p, hsa-miR-744-3p, hsa-miR-30d-5p, hsa-miR-193a-5p, hsa-miR-107, hsa-miR-140-5p, hsa-miR-34a-5p, hsa-miR-1287-5p, hsa-miR-1468-5p, hsa-miR-6727-3p, hsa-miR-299-5p, hsa-miR-1266-3p, hsa-miR-4762-3p, hsa-miR-378a-3p, hsa-miR-6807-5p, hsa-miR-874-5p, hsa-miR-6752-3p, hsa-miR-6886-5p, hsa-miR-146b-3p, hsa-miR-146b-5p, hsa-miR-510-3p, hsa-miR-6813-5p, hsa-miR-6133, hsa-miR-6862-3p, hsa-miR-6879-5p, hsa-miR-1267, hsa-miR-6082, hsa-miR-4423-5p, hsa-miR-4420, hsa-miR-196a-5p | 45 |
| In iPSCs, LogFC>0.3, FDR<0.05 | hsa-miR-1275, hsa-miR-4326, hsa-miR-1273c, hsa-miR-1255a, hsa-miR-7851-3p, hsa-miR-1273a, hsa-miR-28-3p, hsa-miR-4257, hsa-miR-5787, hsa-miR-92b-3p, hsa-miR-572, hsa-miR-1910-5p, hsa-miR-6873-5p, hsa-miR-519d-5p, hsa-miR-4661-5p, hsa-miR-6786-3p, hsa-miR-424-3p, hsa-miR-1343-5p, hsa-miR-4634, hsa-miR-1273g-5p, hsa-miR-4479, hsa-miR-125a-3p, hsa-miR-24-2-5p, hsa-miR-3675-3p, hsa-miR-222-3p, hsa-miR-125a-5p, hsa-miR-6801-3p, hsa-miR-3195, hsa-miR-513a-5p, hsa-miR-518e-3p, hsa-miR-5090, hsa-miR-6086, hsa-miR-6856-3p, hsa-miR-645, hsa-miR-6134, hsa-miR-7155-3p, hsa-miR-1273d, hsa-miR-3617-3p, hsa-miR-570-3p, hsa-miR-4313, hsa-miR-3175, hsa-miR-1539, hsa-miR-4425, hsa-miR-611, hsa-miR-3939, hsa-miR-1-3p, hsa-miR-4494, hsa-miR-593-5p, hsa-miR-4321, hsa-miR-6797-3p, hsa-miR-222-5p, hsa-miR-3529-5p, hsa-miR-1292-3p, hsa-miR-4731-3p, hsa-miR-196b-5p, hsa-miR-3184-3p, hsa-miR-143-3p, hsa-miR-423-5p, hsa-miR-4746-3p, hsa-miR-6878-5p, hsa-miR-4261, hsa-miR-517-5p, hsa-miR-6760-5p, hsa-miR-4681, hsa-miR-3605-3p, hsa-miR-6733-5p, hsa-miR-1324, hsa-miR-30c-2-3p, hsa-miR-6509-5p, hsa-miR-3621, hsa-miR-3150a-5p, hsa-miR-1908-3p, hsa-miR-3119, hsa-miR-219a-5p, hsa-miR-199a-5p, hsa-miR-142-5p, hsa-miR-6751-5p, hsa-miR-615-3p, hsa-miR-548t-3p, hsa-miR-4662b, hsa-miR-181b-5p, hsa-miR-4753-5p, hsa-miR-1323, hsa-miR-208b-3p, hsa-miR-2053, hsa-let-7g-5p, hsa-miR-6815-5p, hsa-miR-4662a-5p, hsa-miR-323a-5p, hsa-miR-6787-3p, hsa-miR-3945, hsa-miR-98-5p, hsa-miR-3689b-3p, hsa-let-7e-5p, hsa-miR-372-5p, hsa-miR-612, hsa-miR-125b-1-3p, hsa-miR-181a-5p, hsa-miR-4435, hsa-miR-4292, hsa-miR-10a-5p, hsa-miR-4303, hsa-miR-219b-3p, hsa-miR-4537, hsa-let-7d-5p, hsa-miR-6716-5p, hsa-miR-3120-3p, hsa-miR-214-3p, hsa-miR-412-3p, hsa-miR-373-5p, hsa-miR-663a, hsa-miR-548ag, hsa-miR-125b-2-3p, hsa-let-7f-5p, hsa-miR-3655, hsa-let-7i-5p, hsa-miR-6862-3p, hsa-miR-214-5p, hsa-miR-2113, hsa-miR-5587-5p, hsa-miR-372-3p, hsa-let-7c-5p, hsa-miR-199a-3p, hsa-miR-371a-3p, hsa-miR-3120-5p, hsa-miR-373-3p, hsa-miR-371b-5p, hsa-miR-371b-3p, hsa-miR-371a-5p, hsa-miR-6891-5p, hsa-let-7b-5p, hsa-miR-4264, hsa-miR-4482-3p | 133 |
| In iPSCs, LogFC<-0.3, FDR<0.05 | hsa-miR-6722-3p, hsa-miR-1304-3p, hsa-miR-6879-5p, hsa-miR-8079, hsa-miR-509-3-5p, hsa-miR-186-3p, hsa-miR-6837-5p, hsa-miR-451b, hsa-miR-3667-5p, hsa-miR-7846-3p, hsa-miR-5589-5p, hsa-miR-6807-5p, hsa-miR-4666b, hsa-miR-5582-3p, hsa-miR-3180-5p, hsa-miR-514a-3p, hsa-miR-5092, hsa-miR-139-5p, hsa-miR-4439, hsa-miR-1185-5p, hsa-miR-33b-3p, hsa-miR-302e, hsa-miR-6717-5p, hsa-miR-412-5p, hsa-miR-4684-3p, hsa-miR-4301, hsa-miR-6867-5p, hsa-miR-1266-3p, hsa-miR-323b-5p, hsa-miR-3651, hsa-miR-508-3p, hsa-miR-4704-3p, hsa-miR-3144-3p, hsa-miR-103a-2-5p, hsa-miR-6506-3p, hsa-miR-770-5p, hsa-miR-944, hsa-miR-208a-5p, hsa-miR-193a-3p, hsa-miR-1206, hsa-miR-4309, hsa-miR-4739, hsa-miR-302c-3p, hsa-miR-624-3p, hsa-miR-4300, hsa-miR-4433b-5p, hsa-miR-548a-3p, hsa-miR-4437, hsa-miR-5588-3p, hsa-miR-3607-3p, hsa-miR-1290, hsa-miR-548ar-5p, hsa-miR-34a-5p, hsa-miR-1249-3p, hsa-miR-367-3p, hsa-miR-766-3p, hsa-miR-516b-3p, hsa-miR-4750-5p, hsa-miR-622, hsa-miR-302b-3p, hsa-miR-1294, hsa-miR-302a-3p, hsa-miR-802, hsa-miR-4520-3p, hsa-miR-2278, hsa-miR-642b-3p, hsa-miR-5579-3p, hsa-miR-302b-5p, hsa-miR-1298-5p, hsa-miR-638, hsa-miR-20a-5p, hsa-miR-302d-3p, hsa-miR-331-3p, hsa-miR-5696, hsa-miR-4682, hsa-miR-378a-5p, hsa-miR-551a, hsa-miR-187-3p | 78 |
| In neurons, LogFC>0.3, FDR<0.05 | hsa-miR-30a-5p, hsa-miR-181d-5p, hsa-miR-181c-5p, hsa-miR-1307-3p, hsa-miR-2110, hsa-miR-671-3p, hsa-miR-15a-5p, hsa-miR-339-3p, hsa-miR-28-5p, hsa-miR-3074-3p, hsa-miR-3938, hsa-miR-99b-3p, hsa-miR-708-3p, hsa-miR-505-5p, hsa-let-7e-3p, hsa-miR-30a-3p, hsa-miR-128-1-5p, hsa-miR-769-3p, hsa-miR-663b, hsa-miR-1307-5p, hsa-miR-3613-5p, hsa-miR-30b-3p, hsa-miR-584-5p, hsa-miR-32-3p, hsa-miR-345-5p, hsa-miR-92b-5p, hsa-miR-3177-5p, hsa-miR-27a-3p, hsa-miR-6746-3p, hsa-miR-1251-5p, hsa-miR-3180-3p, hsa-miR-99a-5p, hsa-miR-574-5p, hsa-let-7f-5p, hsa-miR-99a-3p, hsa-miR-148a-5p, hsa-miR-125b-5p, hsa-miR-4532, hsa-miR-125a-5p, hsa-miR-3181, hsa-miR-181b-3p, hsa-miR-1203, hsa-miR-4531, hsa-miR-4787-3p, hsa-miR-4521, hsa-miR-6798-3p, hsa-miR-548h-5p, hsa-miR-4783-5p, hsa-let-7g-5p, hsa-let-7g-3p, hsa-miR-4707-5p, hsa-miR-21-3p, hsa-miR-125b-2-3p, hsa-miR-34a-3p, hsa-let-7d-3p, hsa-miR-4707-3p, hsa-miR-27a-5p, hsa-miR-3176, hsa-miR-2277-3p, hsa-miR-3683, hsa-miR-20b-5p, hsa-miR-6075, hsa-miR-4281, hsa-miR-1292-3p, hsa-let-7c-5p, hsa-miR-221-5p, hsa-miR-4324, hsa-miR-1915-5p, hsa-miR-4462, hsa-miR-1228-5p, hsa-miR-653-3p, hsa-miR-100-5p, hsa-miR-6724-5p, hsa-miR-106a-5p, hsa-miR-196a-3p, hsa-miR-653-5p, hsa-miR-23a-5p, hsa-miR-222-3p, hsa-miR-34b-3p, hsa-miR-4472, hsa-let-7c-3p, hsa-miR-125b-1-3p, hsa-miR-1915-3p, hsa-miR-302a-3p, hsa-miR-6840-5p, hsa-miR-3190-3p, hsa-miR-489-3p, hsa-miR-122-3p, hsa-miR-1260b, hsa-let-7a-5p, hsa-miR-4681, hsa-miR-214-5p, hsa-miR-499a-3p, hsa-miR-1470, hsa-miR-4510, hsa-miR-302c-3p, hsa-miR-302a-5p, hsa-let-7b-5p, hsa-miR-222-5p, hsa-miR-4455, hsa-miR-100-3p, hsa-miR-302c-5p, hsa-miR-302d-3p, hsa-miR-302b-3p | 104 |
| In neurons, LogFC<-0.3, FDR<0.05 | hsa-miR-196a-5p, hsa-miR-1248, hsa-miR-6851-5p, hsa-miR-6082, hsa-miR-3609, hsa-miR-6894-5p, hsa-miR-3160-3p, hsa-miR-3148, hsa-miR-512-5p, hsa-miR-6501-5p, hsa-miR-6878-5p, hsa-miR-3619-3p, hsa-miR-8087, hsa-miR-6873-5p, hsa-miR-612, hsa-miR-4453, hsa-miR-2117, hsa-miR-1273f, hsa-miR-4452, hsa-miR-3908, hsa-miR-6869-3p, hsa-miR-4307, hsa-miR-5196-3p, hsa-miR-3654, hsa-miR-4499, hsa-miR-5186, hsa-miR-1273h-5p, hsa-miR-8079, hsa-miR-6835-3p, hsa-miR-4426, hsa-miR-378j, hsa-miR-649, hsa-miR-6760-5p, hsa-miR-4277, hsa-miR-4782-5p, hsa-miR-8061, hsa-miR-3617-5p, hsa-miR-5706, hsa-miR-6849-3p, hsa-miR-6893-5p, hsa-miR-616-3p, hsa-miR-5197-5p, hsa-miR-5693, hsa-miR-1273e, hsa-miR-1304-3p, hsa-miR-4509, hsa-miR-6798-5p, hsa-miR-4700-3p, hsa-miR-619-5p, hsa-miR-7110-5p, hsa-miR-519c-5p, hsa-miR-3922-3p, hsa-miR-1250-3p, hsa-miR-938, hsa-miR-4782-3p, hsa-miR-4776-3p, hsa-miR-6876-3p, hsa-miR-5096, hsa-miR-7154-5p, hsa-miR-6823-5p, hsa-miR-4718, hsa-miR-3138, hsa-miR-605-5p, hsa-miR-4311, hsa-miR-6831-5p, hsa-miR-4468, hsa-miR-1285-5p, hsa-miR-606, hsa-miR-5007-3p, hsa-miR-4329, hsa-miR-5684, hsa-miR-153-3p, hsa-miR-1233-5p, hsa-miR-5700, hsa-miR-520d-5p, hsa-miR-5009-5p, hsa-miR-1537-3p, hsa-miR-6822-3p, hsa-miR-6757-5p, hsa-miR-4480, hsa-miR-4672, hsa-miR-7703, hsa-miR-3925-3p, hsa-miR-3667-5p, hsa-miR-4764-5p, hsa-miR-548x-3p, hsa-miR-4494, hsa-miR-6784-5p, hsa-miR-569, hsa-miR-6128, hsa-miR-4693-3p, hsa-miR-4777-3p, hsa-miR-6865-3p, hsa-miR-3916, hsa-miR-3614-3p, hsa-miR-2909, hsa-miR-516a-5p, hsa-miR-1295b-3p, hsa-miR-6778-3p, hsa-miR-6887-5p, hsa-miR-4253, hsa-miR-5584-5p, hsa-miR-657, hsa-miR-3171, hsa-miR-761, hsa-miR-1199-3p, hsa-miR-3133, hsa-miR-6085, hsa-miR-6838-3p, hsa-miR-182-3p, hsa-miR-3156-5p, hsa-miR-3618, hsa-miR-4525, hsa-miR-203a-3p, hsa-miR-3653-5p, hsa-miR-4677-5p, hsa-miR-4778-5p, hsa-miR-6773-5p, hsa-miR-1224-5p, hsa-miR-1273d, hsa-miR-4529-5p, hsa-miR-6828-5p, hsa-miR-509-3p, hsa-miR-6818-3p, hsa-miR-6740-3p, hsa-miR-1273h-3p, hsa-miR-23c, hsa-miR-203b-5p, hsa-miR-1273g-3p, hsa-miR-3159, hsa-miR-4535, hsa-miR-153-5p, hsa-miR-6879-5p, hsa-miR-146a-5p, hsa-miR-4644, hsa-miR-4434, hsa-miR-3942-3p, hsa-miR-6736-3p, hsa-miR-5588-5p, hsa-miR-378a-5p, hsa-miR-5095, hsa-miR-1910-3p, hsa-miR-6079, hsa-miR-516b-5p, hsa-miR-2681-3p, hsa-miR-3607-5p, hsa-miR-2276-5p, hsa-miR-6827-3p, hsa-miR-6852-3p, hsa-miR-6891-3p, hsa-miR-3168, hsa-miR-4749-5p, hsa-miR-676-5p, hsa-miR-4742-3p, hsa-miR-6746-5p, hsa-miR-4668-5p, hsa-miR-4439, hsa-miR-6866-3p, hsa-miR-4755-3p, hsa-miR-5689, hsa-miR-510-5p, hsa-miR-5100, hsa-miR-5585-3p, hsa-miR-4430, hsa-miR-5193, hsa-miR-1976, hsa-miR-3653-3p, hsa-miR-1273a | 168 |

Supplementary Table 5 Predicted miRNAs potentially binding to circFTO

| miRNA | Total Score | Total Energy | Max Score | Max Energy | Strand |
| --- | --- | --- | --- | --- | --- |
| hsa-miR-638 | 2397 | -469.63 | 137 | -32.73 | 684 |
| hsa-miR-1304-3p | 3163 | -380.22 | 171 | -32.24 | 912 |
| hsa-miR-6722-3p | 5659 | -999.08 | 155 | -31.69 | 2118 |
| hsa-miR-4739 | 5885 | -1028.79 | 163 | -30.89 | 1769 |
| hsa-miR-3180-5p | 4281 | -548.97 | 134 | -29.57 | 1143 |
| hsa-miR-4682 | 5603 | -773.13 | 136 | -28.92 | 1666 |
| hsa-miR-3651 | 3308 | -474.76 | 176 | -28.7 | 1301 |
| hsa-miR-187-3p | 4456 | -585.86 | 144 | -28.53 | 130 |
| hsa-miR-6879-5p | 6378 | -1006.57 | 155 | -27.89 | 2427 |
| hsa-miR-367-3p | 3547 | -358.05 | 156 | -27.63 | 315 |
| hsa-miR-7846-3p | 3436 | -458.35 | 135 | -27.52 | 2520 |
| hsa-miR-302a-3p | 5238 | -547.19 | 148 | -26.98 | 278 |
| hsa-miR-4439 | 4474 | -540.73 | 156 | -26.96 | 1447 |
| hsa-miR-551a | 1304 | -170.93 | 122 | -26.91 | 562 |
| hsa-miR-6807-5p | 5358 | -739.89 | 156 | -26.62 | 2283 |
| hsa-miR-4750-5p | 2133 | -414.4 | 124 | -26.54 | 1789 |
| hsa-miR-33b-3p | 4401 | -608.88 | 144 | -26.14 | 677 |
| hsa-miR-766-3p | 3748 | -455.52 | 151 | -25.94 | 764 |
| hsa-miR-378a-5p | 2215 | -326.92 | 132 | -25.93 | 336 |
| hsa-miR-2278 | 6918 | -841.88 | 143 | -24.8 | 1033 |
| hsa-miR-770-5p | 5229 | -655.53 | 140 | -24.67 | 778 |
| hsa-miR-323b-5p | 2826 | -408.73 | 128 | -24.48 | 1134 |
| hsa-miR-5589-5p | 4490 | -632.98 | 158 | -24.45 | 1984 |
| hsa-miR-622 | 6445 | -835.74 | 151 | -24.36 | 661 |
| hsa-miR-193a-3p | 3026 | -367.11 | 139 | -24.25 | 255 |
| hsa-miR-302c-3p | 5492 | -625.85 | 148 | -24.12 | 311 |
| hsa-miR-6837-5p | 4574 | -664.86 | 142 | -24.08 | 2345 |
| hsa-miR-34a-5p | 5258 | -674.16 | 142 | -24.03 | 117 |
| hsa-miR-4437 | 3644 | -461.34 | 137 | -23.81 | 1443 |
| hsa-miR-8079 | 4076 | -464.2 | 141 | -23.6 | 2565 |
| hsa-miR-508-3p | 5106 | -548.6 | 136 | -23.39 | 543 |
| hsa-miR-6867-5p | 3802 | -488.43 | 143 | -23.23 | 2403 |
| hsa-miR-3144-3p | 3219 | -334.24 | 139 | -23.18 | 1089 |
| hsa-miR-4433b-5p | 3232 | -396.5 | 140 | -23.14 | 2512 |
| hsa-miR-6717-5p | 2140 | -303.27 | 120 | -23.11 | 2109 |
| hsa-miR-302d-3p | 5172 | -514.93 | 140 | -23 | 313 |
| hsa-miR-1266-3p | 4167 | -484.36 | 149 | -22.37 | 945 |
| hsa-miR-642b-3p | 5198 | -583.65 | 138 | -22.21 | 1409 |
| hsa-miR-331-3p | 2476 | -315.17 | 140 | -22.02 | 368 |
| hsa-miR-302b-3p | 4272 | -433.62 | 144 | -21.78 | 309 |
| hsa-miR-208a-5p | 4524 | -491.81 | 132 | -21.56 | 97 |
| hsa-miR-139-5p | 4449 | -485.01 | 129 | -21.52 | 107 |
| hsa-miR-5582-3p | 4501 | -389.47 | 154 | -21.19 | 1967 |
| hsa-miR-1298-5p | 2609 | -296.74 | 124 | -21.12 | 771 |
| hsa-miR-1290 | 4802 | -533.31 | 130 | -21.11 | 900 |
| hsa-miR-103a-2-5p | 4481 | -504.99 | 134 | -21.08 | 79 |
| hsa-miR-624-3p | 5281 | -513.17 | 157 | -21.03 | 664 |
| hsa-miR-4309 | 4371 | -477.78 | 159 | -20.51 | 1199 |
| hsa-miR-4666b | 4605 | -431.62 | 150 | -19.74 | 2008 |
| hsa-miR-4301 | 2300 | -278.07 | 154 | -19.71 | 1190 |
| hsa-miR-1294 | 6491 | -767.35 | 142 | -19.67 | 904 |
| hsa-miR-3667-5p | 2795 | -328.04 | 147 | -19.61 | 1317 |
| hsa-miR-186-3p | 5186 | -628.19 | 149 | -19.45 | 249 |
| hsa-miR-4684-3p | 3962 | -444.88 | 128 | -19.45 | 1669 |
| hsa-miR-4520-3p | 4090 | -446.46 | 143 | -19.45 | 1549 |
| hsa-miR-20a-5p | 6774 | -680.53 | 148 | -19.37 | 28 |
| hsa-miR-4704-3p | 4836 | -484.32 | 150 | -18.9 | 1703 |
| hsa-miR-412-5p | 1359 | -170.52 | 125 | -18.84 | 412 |
| hsa-miR-516b-3p | 3293 | -366.01 | 130 | -18.73 | 505 |
| hsa-miR-514a-3p | 3968 | -400.45 | 130 | -18.6 | 549 |
| hsa-miR-944 | 2652 | -216.03 | 133 | -18.6 | 849 |
| hsa-miR-1249-3p | 1587 | -159.12 | 124 | -18.53 | 924 |
| hsa-miR-5588-3p | 4200 | -422.19 | 132 | -18.47 | 1983 |
| hsa-miR-5696 | 5175 | -471.05 | 152 | -18.28 | 2012 |
| hsa-miR-1185-5p | 4211 | -389.03 | 137 | -18.18 | 756 |
| hsa-miR-5092 | 2518 | -303.07 | 128 | -17.99 | 1924 |
| hsa-miR-6506-3p | 2920 | -324.01 | 134 | -17.96 | 2085 |
| hsa-miR-509-3-5p | 4555 | -511.58 | 127 | -17.07 | 834 |
| hsa-miR-4300 | 2835 | -337.93 | 127 | -16.83 | 1193 |
| hsa-miR-1206 | 3943 | -357.5 | 142 | -16.58 | 886 |
| hsa-miR-548a-3p | 3614 | -301.19 | 149 | -15.72 | 609 |
| hsa-miR-5579-3p | 3457 | -334.09 | 135 | -15.09 | 1957 |
| hsa-miR-302e | 2025 | -179.91 | 129 | -14.77 | 955 |
| hsa-miR-302b-5p | 4051 | -354.23 | 144 | -14.59 | 308 |
| hsa-miR-451b | 4902 | -439.86 | 150 | -14.4 | 1741 |
| hsa-miR-802 | 2891 | -250.38 | 134 | -13.37 | 768 |
| hsa-miR-548ar-5p | 3934 | -320.6 | 138 | -12.74 | 1952 |

Supplementary Table 6 Predicted targets potentially binding to miR-187-3p

| geneName | clipExpNum | TDMDScore | phyloP | pancancerNum |
| --- | --- | --- | --- | --- |
| TMSB10 | 41 | 1.7308 | 0.85 | 9 |
| DYRK2 | 24 | 1.1115 | 4.164 | 11 |
| FRAT2 | 18 | 0.6175 | 0.203 | 9 |
| GIPC1 | 17 | 0.7273 | -0.639 | 4 |
| MBNL1 | 16 | 1.2594 | 4.411 | 6 |
| EEF2 | 11 | 0.7317 | -0.215 | 8 |
| KRT8 | 10 | 0.9633 | 3.107 | 7 |
| SPTBN1 | 8 | 1.2305 | 5.079 | 11 |
| SMAD1 | 8 | 0.9844 | 2.731 | 5 |
| FLNC | 7 | 0.9638 | 0.602 | 10 |
| ARHGDIA | 7 | 0.9057 | -0.231 | 7 |
| ADNP | 7 | 0.5765 | 1.345 | 6 |
| CAPZB | 6 | 1.1086 | 2.208 | 8 |
| FLNB | 6 | 1.0687 | 0.194 | 8 |
| DAB2 | 6 | 1.0333 | 0.718 | 6 |
| S100A4 | 6 | 0.868 | -0.22 | 6 |
| DUS1L | 6 | 0.5654 | 0.119 | 9 |
| IGF1R | 5 | 1.2971 | 0.167 | 6 |
| JUN | 5 | 1.2742 | 4.376 | 3 |
| SOX4 | 5 | 1.2328 | 1.346 | 7 |
| RPP25 | 5 | 1.0044 | -0.512 | 9 |
| STAU1 | 5 | 0.9401 | 0.673 | 3 |
| ZNF652 | 5 | 0.9091 | -1.187 | 9 |
| AKIRIN1 | 5 | 0.7959 | 0.606 | 4 |
| AKAP11 | 5 | 0.5843 | 0.052 | 7 |
| NFKBIZ | 5 | 0.5203 | 1.883 | 6 |
| ANP32E | 4 | 2.1376 | 0.293 | 9 |
| PSMD2 | 4 | 1.5642 | 0.61 | 4 |
| MPZL1 | 4 | 1.3596 | 1.31 | 5 |
| CAVIN1 | 4 | 1.1951 | -0.105 | 7 |
| HIPK3 | 4 | 1.0188 | 6.118 | 6 |
| PPAT | 4 | 1.0055 | -0.01 | 9 |
| TFF1 | 4 | 0.9922 | -1.13 | 4 |
| LDLR | 4 | 0.9346 | -0.268 | 5 |
| ACTR1A | 4 | 0.6563 | 0.216 | 3 |
| INSR | 4 | 0.5924 | 0.227 | 9 |
| GSR | 4 | 0.0075 | -0.561 | 6 |
| SLC38A1 | 3 | 2.0136 | 0.503 | 4 |
| LRFN1 | 3 | 1.5414 | 2.411 | 9 |
| SLC6A6 | 3 | 1.538 | 0.033 | 3 |
| CHD4 | 3 | 1.4705 | 0.891 | 5 |
| BCL6 | 3 | 1.3341 | 4.98 | 7 |
| WNT5A | 3 | 1.2742 | -0.348 | 3 |
| FOXF1 | 3 | 1.2406 | 3.54 | 7 |
| BCL10 | 3 | 1.2261 | -0.246 | 9 |
| TMEM50A | 3 | 1.2039 | -0.289 | 6 |
| TSPAN5 | 3 | 1.1977 | 0.054 | 4 |
| SLC22A23 | 3 | 1.1928 | -0.663 | 2 |
| UCHL1 | 3 | 1.1308 | 0.346 | 6 |
| MRPL27 | 3 | 1.1086 | -0.109 | 4 |
| GANAB | 3 | 1.1019 | 1.497 | 10 |
| SLC7A1 | 3 | 1.0932 | -0.123 | 9 |
| SPARC | 3 | 1.0321 | -0.125 | 9 |
| NDRG3 | 3 | 1.0133 | 2.728 | 5 |
| RNF34 | 3 | 1.0133 | -0.483 | 6 |
| NR2F6 | 3 | 1.0066 | 0.226 | 6 |
| TIMP2 | 3 | 1.0066 | 0.019 | 9 |
| NPLOC4 | 3 | 0.9922 | -0.656 | 4 |
| PRR14L | 3 | 0.9734 | 0.329 | 4 |
| MTUS1 | 3 | 0.9467 | -0.225 | 7 |
| ARHGAP35 | 3 | 0.9091 | -0.322 | 8 |
| SEPTIN4 | 3 | 0.8891 | 3.395 | 5 |
| NBPF15 | 3 | 0.8558 | -0.755 | 6 |
| TSPAN3 | 3 | 0.8037 | -0.282 | 8 |
| ZNF384 | 3 | 0.7206 | 2.579 | 8 |
| SHROOM3 | 3 | 0.7128 | 0.197 | 8 |
| TFRC | 3 | 0.5765 | 0.618 | 6 |
| PTPN11 | 3 | 0.307 | -2.21 | 5 |
| ZC3H4 | 2 | 1.9315 | 2.584 | 6 |
| RASD1 | 2 | 1.8474 | 2.417 | 1 |
| HMGA2 | 2 | 1.5724 | 1.435 | 6 |
| ADCY1 | 2 | 1.568 | -0.564 | 3 |
| CDCA4 | 2 | 1.5542 | -0.051 | 9 |
| DHCR24 | 2 | 1.5188 | -0.159 | 6 |
| CALM1 | 2 | 1.5035 | 1.948 | 4 |
| LASP1 | 2 | 1.496 | -0.676 | 14 |
| STAT3 | 2 | 1.4781 | -0.189 | 7 |
| RBM38 | 2 | 1.4398 | -0.186 | 6 |
| NECTIN1 | 2 | 1.4146 | -0.459 | 3 |
| ALDH3B2 | 2 | 1.4013 | 0.209 | 2 |
| DTYMK | 2 | 1.388 | 2.123 | 8 |
| SMARCD2 | 2 | 1.3503 | 0.562 | 9 |
| NDRG1 | 2 | 1.3248 | -0.394 | 6 |
| TMEM250 | 2 | 1.3214 | -0.887 | 6 |
| PHF13 | 2 | 1.3197 | 0.256 | 4 |
| SEMA4C | 2 | 1.3049 | -0.168 | 4 |
| C1S | 2 | 1.2782 | -0.455 | 8 |
| XPO6 | 2 | 1.2782 | 0.004 | 8 |
| ABLIM1 | 2 | 1.2672 | -0.565 | 9 |
| DGCR8 | 2 | 1.2039 | -1.146 | 4 |
| CD276 | 2 | 1.1928 | 0.296 | 6 |
| DNAJC16 | 2 | 1.1885 | -0.481 | 10 |
| MFN2 | 2 | 1.1763 | -0.325 | 5 |
| ELOVL5 | 2 | 1.1474 | 0.979 | 9 |
| CD93 | 2 | 1.1459 | 0.183 | 8 |
| MDM2 | 2 | 1.1308 | 4.259 | 3 |
| SMIM13 | 2 | 1.1097 | 0.207 | 5 |
| UBXN4 | 2 | 1.1086 | 0.218 | 9 |
| SLC6A8 | 2 | 1.1019 | -0.747 | 4 |
| PLOD3 | 2 | 1.0976 | -0.212 | 8 |
| CCNL2 | 2 | 1.0941 | 0.24 | 8 |
| JRK | 2 | 1.0898 | -0.287 | 11 |
| PDXK | 2 | 1.0831 | -0.687 | 7 |
| NTPCR | 2 | 1.0753 | -0.855 | 3 |
| ELAVL3 | 2 | 1.0565 | 0.16 | 5 |
| SACM1L | 2 | 1.0487 | -0.445 | 7 |
| WIPI1 | 2 | 1.0399 | 5.267 | 6 |
| UPF1 | 2 | 1.0321 | -0.729 | 5 |
| BTBD9 | 2 | 1.0133 | -0.537 | 5 |
| PABPN1 | 2 | 1.011 | 2.437 | 2 |
| BCL2L2-PABPN1 | 2 | 1.011 | 2.437 | 7 |
| USB1 | 2 | 1.0023 | -0.82 | 2 |
| BTBD2 | 2 | 1 | -0.655 | 6 |
| NECTIN1 | 2 | 0.9756 | 0.904 | 3 |
| BCL2L11 | 2 | 0.9707 | -1.534 | 7 |
| CBFA2T3 | 2 | 0.9401 | -0.614 | 5 |
| SEC24C | 2 | 0.9268 | 5.813 | 3 |
| KDM2A | 2 | 0.9235 | 0.202 | 4 |
| RPL35 | 2 | 0.9213 | 4.931 | 9 |
| PDXK | 2 | 0.9157 | 0.11 | 7 |
| DUSP3 | 2 | 0.8703 | -0.553 | 9 |
| CHMP2B | 2 | 0.8037 | -0.109 | 6 |
| MANEAL | 2 | 0.7916 | 4.655 | 6 |
| CTBP1 | 2 | 0.7727 | -1.461 | 7 |
| CAVIN1 | 2 | 0.7594 | 0.316 | 7 |
| UBE2E1 | 2 | 0.7583 | 1.812 | 6 |
| SEC61A1 | 2 | 0.695 | -0.812 | 5 |
| GOT1 | 2 | 0.6943 | -0.164 | 8 |
| SLBP | 2 | 0.6818 | -0.423 | 5 |
| ATP6V0E2 | 2 | 0.6752 | -0.571 | 7 |
| RBM22 | 2 | 0.663 | 0.311 | 10 |
| GALNT11 | 2 | 0.6563 | -0.937 | 6 |
| CCNF | 2 | 0.6253 | -0.485 | 6 |
| AC012213.5 | 2 | 0.6219 | 0.114 | 0 |
| SLC25A32 | 2 | 0.6219 | 0.114 | 5 |
| ODC1 | 2 | 0.5843 | 2.612 | 7 |
| FOXK1 | 2 | 0.5802 | 0.242 | 8 |
| MAPK1 | 2 | 0.5654 | -0.404 | 2 |
| MAFF | 2 | 0.5576 | -0.336 | 3 |
| ABL2 | 2 | 0.52 | -0.369 | 8 |
| INCENP | 2 | 0.3836 | 0.024 | 4 |
| THSD4 | 2 | 0.2287 | 0.5 | 3 |
| CC2D1B | 2 | -0.0174 | 0.165 | 4 |
| ACVRL1 | 2 | -0.2794 | -0.135 | 12 |
| ERBB2 | 1 | 1.7763 | 2.922 | 7 |
| CDCP1 | 1 | 1.7352 | 2.41 | 6 |
| FADS1 | 1 | 1.7308 | 0.39 | 10 |
| FADS1 | 1 | 1.7308 | -0.278 | 10 |
| B4GALT7 | 1 | 1.599 | 0.67 | 5 |
| CYTH3 | 1 | 1.5987 | -1.64 | 7 |
| ARHGAP33 | 1 | 1.5669 | 0.856 | 10 |
| MAP2 | 1 | 1.5536 | 2.019 | 7 |
| MAP1B | 1 | 1.5165 | -0.427 | 6 |
| AC026464.6 | 1 | 1.5003 | 0.275 | 0 |
| COG8 | 1 | 1.5003 | 0.275 | 6 |
| PDF | 1 | 1.5003 | 0.275 | 7 |
| KCNH2 | 1 | 1.4771 | 0.44 | 5 |
| MNT | 1 | 1.4438 | -0.606 | 2 |
| ATP11B | 1 | 1.425 | 0.534 | 5 |
| CCND1 | 1 | 1.4212 | 1.513 | 5 |
| POU2F2 | 1 | 1.3769 | 1.358 | 10 |
| FLRT2 | 1 | 1.3714 | 2.135 | 7 |
| ELAVL3 | 1 | 1.3503 | -0.48 | 5 |
| LMTK2 | 1 | 1.3448 | -0.119 | 8 |
| PTTG1IP | 1 | 1.3341 | -0.572 | 9 |
| DUS1L | 1 | 1.3315 | -0.491 | 9 |
| RIMKLB | 1 | 1.3263 | 4.041 | 5 |
| HDAC4 | 1 | 1.3263 | 0.027 | 8 |
| CMTM4 | 1 | 1.3259 | -0.262 | 7 |
| ABI2 | 1 | 1.3214 | -0.858 | 12 |
| ATP11A | 1 | 1.2838 | -0.645 | 6 |
| NIPAL1 | 1 | 1.282 | 0.281 | 3 |
| SEMA6B | 1 | 1.276 | -0.515 | 7 |
| AC010463.1 | 1 | 1.276 | 3.857 | 1 |
| BABAM1 | 1 | 1.276 | 3.857 | 4 |
| FBXO31 | 1 | 1.2594 | -0.487 | 6 |
| TTYH3 | 1 | 1.2539 | -1.037 | 6 |
| KLHL21 | 1 | 1.2472 | -0.703 | 6 |
| A2M | 1 | 1.2472 | -0.465 | 7 |
| WDR5 | 1 | 1.2472 | -0.874 | 11 |
| ONECUT3 | 1 | 1.2472 | -0.194 | 4 |
| CSNK1D | 1 | 1.2406 | 1.924 | 5 |
| PSMD1 | 1 | 1.235 | 5.329 | 5 |
| MYD88 | 1 | 1.2183 | 0.403 | 5 |
| TSPAN9 | 1 | 1.1951 | -0.378 | 3 |
| SUPT16H | 1 | 1.1951 | 1.342 | 6 |
| BCL9L | 1 | 1.1951 | -0.337 | 7 |
| RHBDF1 | 1 | 1.1951 | -0.27 | 7 |
| SERPINE1 | 1 | 1.1928 | 2.138 | 9 |
| ERBB2 | 1 | 1.1807 | 3.385 | 7 |
| NDST1 | 1 | 1.1696 | 0.194 | 6 |
| LAMB1 | 1 | 1.1375 | 4.147 | 5 |
| PLA2G12A | 1 | 1.1364 | 0.084 | 8 |
| CCM2 | 1 | 1.1268 | 0.025 | 5 |
| FZD1 | 1 | 1.1042 | -0.501 | 6 |
| FSTL3 | 1 | 1.1042 | -0.587 | 12 |
| ATP11A | 1 | 1.1019 | -0.303 | 6 |
| DOT1L | 1 | 1.0976 | 0.043 | 9 |
| PIP5K1C | 1 | 1.0941 | -0.797 | 3 |
| ZNF516 | 1 | 1.0898 | 0.572 | 2 |
| RNF24 | 1 | 1.0854 | 0.007 | 7 |
| NRARP | 1 | 1.0831 | -0.348 | 9 |
| BCL2L2 | 1 | 1.0623 | 0.355 | 7 |
| TTL | 1 | 1.0609 | 0.487 | 5 |
| ZKSCAN8 | 1 | 1.0565 | -0.459 | 7 |
| PDPR | 1 | 1.0399 | -0.214 | 5 |
| KIF1A | 1 | 1.0333 | -1.402 | 6 |
| RYR2 | 1 | 1.0255 | 0.575 | 3 |
| ATP2B4 | 1 | 1.0133 | 1.091 | 5 |
| ACHE | 1 | 1.0066 | 3.592 | 8 |
| TM9SF4 | 1 | 1.0023 | -0.304 | 4 |
| DCLK1 | 1 | 0.9945 | 0.486 | 3 |
| CRMP1 | 1 | 0.9945 | 1.408 | 4 |
| PROSER2 | 1 | 0.9922 | 0.313 | 4 |
| TRIP11 | 1 | 0.9667 | -0.061 | 5 |
| ADCY9 | 1 | 0.9545 | 0.795 | 10 |
| GRIA2 | 1 | 0.9545 | 6.198 | 5 |
| MYO9B | 1 | 0.9424 | 0.018 | 5 |
| EPN2 | 1 | 0.9331 | -0.198 | 7 |
| PTMA | 1 | 0.9279 | 0.827 | 10 |
| SIPA1L3 | 1 | 0.9224 | -0.275 | 9 |
| ACVR2B | 1 | 0.8969 | 2.528 | 7 |
| ATP8A1 | 1 | 0.8868 | -0.144 | 9 |
| ADARB1 | 1 | 0.8825 | -1.018 | 4 |
| GNA11 | 1 | 0.8492 | 0.111 | 8 |
| ARMC1 | 1 | 0.8437 | 0.781 | 7 |
| BTN3A2 | 1 | 0.8437 | -1.21 | 9 |
| ST3GAL4 | 1 | 0.8292 | 0.324 | 4 |
| ICMT | 1 | 0.8226 | -1.303 | 3 |
| ZBED4 | 1 | 0.806 | 0.802 | 7 |
| NT5E | 1 | 0.7916 | 0.037 | 7 |
| NPTXR | 1 | 0.7872 | -1.58 | 4 |
| UBE2L3 | 1 | 0.786 | 3.213 | 2 |
| YARS1 | 1 | 0.7805 | 0.178 | 10 |
| VPS13C | 1 | 0.7771 | -0.652 | 6 |
| CYB5RL | 1 | 0.7771 | -1.229 | 7 |
| ZHX3 | 1 | 0.7727 | -0.598 | 10 |
| SLC23A2 | 1 | 0.7583 | -0.803 | 6 |
| MOB2 | 1 | 0.7505 | -0.235 | 1 |
| ETS1 | 1 | 0.7482 | -0.038 | 12 |
| TRIB2 | 1 | 0.7084 | 3.463 | 2 |
| FAM89A | 1 | 0.7084 | 0.333 | 4 |
| SYNCRIP | 1 | 0.7029 | 4.901 | 7 |
| ARRDC3 | 1 | 0.7018 | 2.174 | 7 |
| STRADA | 1 | 0.694 | -0.696 | 6 |
| ABCC3 | 1 | 0.6862 | -0.687 | 13 |
| TMCC2 | 1 | 0.6833 | 5.067 | 1 |
| ANO7 | 1 | 0.6563 | -1.489 | 5 |
| ZNF74 | 1 | 0.6485 | -0.063 | 6 |
| NBPF10 | 1 | 0.6364 | -0.13 | 6 |
| LMTK2 | 1 | 0.6364 | -0.465 | 8 |
| ZNF343 | 1 | 0.6323 | 0.44 | 6 |
| GET1 | 1 | 0.63 | -1.182 | 4 |
| CDKN2A | 1 | 0.6175 | 4.189 | 4 |
| ASIC1 | 1 | 0.6153 | 3.687 | 3 |
| JAG1 | 1 | 0.5576 | 0.355 | 7 |
| HELZ | 1 | 0.5533 | 2.031 | 8 |
| STMN1 | 1 | 0.5388 | -0.294 | 8 |
| ADCY9 | 1 | 0.5322 | 0.361 | 10 |
| ADD2 | 1 | 0.5266 | 1.673 | 6 |
| MTR | 1 | 0.4934 | -0.244 | 7 |
| LRRN1 | 1 | 0.4934 | 2.565 | 7 |
| CYB5A | 1 | 0.4867 | 0.343 | 8 |
| STX16 | 1 | 0.4544 | 0.167 | 7 |
| ATP11A | 1 | 0.4461 | -0.831 | 6 |
| PDK1 | 1 | 0.4089 | 0.471 | 7 |
| HMOX1 | 1 | 0.3836 | 0.141 | 5 |
| API5 | 1 | 0.3836 | 2.256 | 8 |
| EVL | 1 | 0.3651 | 2.367 | 5 |
| MAP3K20 | 1 | 0.3651 | 0.461 | 8 |
| ABCA3 | 1 | 0.3419 | 0.318 | 10 |
| CD47 | 1 | 0.3315 | 1.879 | 7 |
| SCARB2 | 1 | 0.3153 | -0.842 | 7 |
| STAU2 | 1 | 0.3072 | 0.075 | 8 |
| CCNL2 | 1 | 0.2992 | -0.552 | 8 |
| CERCAM | 1 | 0.2927 | 3.599 | 8 |
| UNC13A | 1 | 0.2487 | -1.213 | 6 |
| SPRY1 | 1 | 0.2406 | 3.92 | 5 |
| PDZRN3 | 1 | 0.2134 | 2.062 | 4 |
| LIPA | 1 | 0.1895 | -0.342 | 6 |
| AGO1 | 1 | 0.1578 | 3.244 | 7 |
| CGA | 1 | 0.0237 | -0.27 | 7 |
| PCBD2 | 1 | 0.0214 | -0.148 | 11 |
| ANGPTL2 | 1 | 0.0107 | 1.643 | 7 |
| MARCKS | 1 | 0.0049 | -0.404 | 7 |
| CDC42BPA | 1 | -0.0139 | -0.664 | 4 |
| VRK1 | 1 | -0.0327 | -0.716 | 5 |
| RBFOX2 | 1 | -0.0672 | 1.325 | 6 |
| LINGO1 | 1 | -0.2146 | -0.101 | 6 |
| WWC3 | 1 | -0.3775 | 1.106 | 6 |
| HDAC4 | 0 | 1.5336 | 0.639 | 8 |
| CYB5RL | 0 | 1.4977 | -0.246 | 7 |
| SLC6A6 | 0 | 1.4545 | 1.923 | 3 |
| MNT | 0 | 1.3836 | 1.166 | 2 |
| KCNH2 | 0 | 1.3425 | 2.033 | 5 |
| CBFA2T3 | 0 | 1.3385 | 5.653 | 5 |
| ONECUT3 | 0 | 1.3237 | -0.057 | 4 |
| PLA2G12A | 0 | 1.3049 | 4.102 | 8 |
| TSPAN9 | 0 | 1.2738 | -1.101 | 3 |
| BTBD9 | 0 | 1.2328 | -0.891 | 5 |
| CHMP2B | 0 | 1.2328 | -0.126 | 6 |
| PDXK | 0 | 1.2039 | -0.064 | 7 |
| BTBD9 | 0 | 1.174 | 0.163 | 5 |
| CD276 | 0 | 1.154 | 2.592 | 6 |
| CD276 | 0 | 1.154 | 2.639 | 6 |
| ONECUT3 | 0 | 1.1497 | -0.496 | 4 |
| ATP2B4 | 0 | 1.0909 | 0.489 | 5 |
| TSPAN3 | 0 | 1.0565 | -0.238 | 8 |
| INSR | 0 | 1.0188 | -0.038 | 9 |
| DOT1L | 0 | 1 | -0.288 | 9 |
| ABL2 | 0 | 0.9778 | -0.5 | 8 |
| CYB5RL | 0 | 0.9545 | -0.532 | 7 |
| PIP5K1C | 0 | 0.9467 | -0.497 | 3 |
| CTBP1 | 0 | 0.9334 | -1.781 | 7 |
| ACVR2B | 0 | 0.857 | 0.236 | 7 |
| ADCY1 | 0 | 0.8492 | -0.314 | 3 |
| NPTXR | 0 | 0.8182 | 0.667 | 4 |
| CTBP1 | 0 | 0.7727 | -0.466 | 7 |
| CTBP1 | 0 | 0.7727 | -1.518 | 7 |
| CTBP1 | 0 | 0.7727 | -1.21 | 7 |
| CTBP1 | 0 | 0.7727 | -2.747 | 7 |
| CTBP1 | 0 | 0.7727 | -1.586 | 7 |
| CTBP1 | 0 | 0.7727 | -0.79 | 7 |
| CTBP1 | 0 | 0.7727 | -1.422 | 7 |
| CTBP1 | 0 | 0.7727 | -1.138 | 7 |
| CTBP1 | 0 | 0.7727 | -1.481 | 7 |
| CTBP1 | 0 | 0.7727 | -0.433 | 7 |
| CMTM4 | 0 | 0.7583 | -0.592 | 7 |
| JRK | 0 | 0.7473 | -1.01 | 11 |
| ABL2 | 0 | 0.7395 | -0.324 | 8 |
| ADCY1 | 0 | 0.7317 | -0.392 | 3 |
| KIF1A | 0 | 0.7206 | -0.31 | 6 |
| TRIB2 | 0 | 0.7018 | 0.543 | 2 |
| ZNF516 | 0 | 0.6963 | 0.421 | 2 |
| FOXK1 | 0 | 0.6752 | 0.813 | 8 |
| POU2F2 | 0 | 0.6112 | -0.157 | 10 |
| UNC13A | 0 | 0.5576 | 1.118 | 6 |
| ATP11A | 0 | 0.5078 | -0.522 | 6 |
| ATP11A | 0 | 0.5078 | -0.139 | 6 |
| ATP11A | 0 | 0.5078 | 0.175 | 6 |
| BTBD9 | 0 | 0.4812 | -0.85 | 5 |
| THSD4 | 0 | 0.388 | -0.042 | 3 |
| THSD4 | 0 | 0.251 | -0.338 | 3 |
| ATP6V0E2 | 0 | 0.1911 | 0.031 | 7 |
| WWC3 | 0 | 0.1413 | 0.29 | 6 |
